# Glycogen Metabolism in Candida albicans Impacts Fitness and Virulence during Vulvovaginal and Invasive Candidiasis

**DOI:** 10.1128/mbio.00046-23

**Published:** 2023-02-22

**Authors:** Jian Miao, Jessica Regan, Chun Cai, Glen E. Palmer, David L. Williams, Michael D. Kruppa, Brian M. Peters

**Affiliations:** a Pharmaceutical Sciences Program, College of Graduate Health Sciences, University of Tennessee Health Science Center, Memphis, Tennessee, USA; b Department of Clinical Pharmacy and Translational Science, College of Pharmacy, University of Tennessee Health Science Center, Memphis, Tennessee, USA; c Department of Microbiology, Immunology, and Biochemistry, College of Medicine, University of Tennessee Health Science Center, Memphis, Tennessee, USA; d Department of Surgery, Quillen College of Medicine, East Tennessee State University, Johnson City, Tennessee, USA; e Center of Excellence in Inflammation, Infectious Disease, and Immunity, East Tennessee State University, Johnson City, Tennessee, USA; f Department of Biomedical Sciences, Quillen College of Medicine, East Tennessee State University, Johnson City, Tennessee, USA; Texas Christian University

**Keywords:** *Candida albicans*, glycogen, metabolism, vulvovaginal, candidiasis, Candida

## Abstract

The polymorphic fungus Candida albicans remains a leading cause of both invasive and superficial mycoses, including vulvovaginal candidiasis (VVC). Metabolic plasticity, including carbohydrate catabolism, confers fitness advantages at anatomical site-specific host niches. C. albicans possesses the capacity to accumulate and store carbohydrates as glycogen and can consume intracellular glycogen stores when nutrients become limited. In the vaginal environment, estrogen promotes epithelial glycogen accumulation and C. albicans colonization. However, whether these factors are mechanistically linked is unexplored. Here, we characterized the glycogen metabolism pathways in C. albicans and investigated whether these impact the long-term survival of C. albicans, both *in vitro* and *in vivo* during murine VVC, or virulence during systemic infection. SC5314 and 6 clinical isolates demonstrated impaired growth when glycogen was used as the sole carbon source, suggesting that environmental glycogen acquisition is limited. The genetic deletion and complementation of key genes involved in glycogen metabolism in Saccharomyces cerevisiae confirmed that *GSY1* and *GLC3*, as well as *GPH1* and *GDB1*, are essential for glycogen synthesis and catabolism in C. albicans, respectively. Potential compensatory roles for a glucoamylase encoded by *SGA1* were also explored. Competitive survival assays revealed that *gsy1Δ/Δ*, *gph1*Δ/Δ, and *gph1Δ/Δ sga1Δ/Δ* mutants exhibited long-term survival defects *in vitro* under starvation conditions and *in vivo* during vaginal colonization. A complete inability to catabolize glycogen (*gph1Δ/Δ sga1Δ/Δ*) also rendered C. albicans significantly less virulent during disseminated infections. This is the first study fully validating the glycogen metabolism pathways in C. albicans, and the results further suggest that intracellular glycogen catabolism positively impacts the long-term fitness of C. albicans in nutrient deficient environments and is important for full virulence.

## INTRODUCTION

The polymorphic human fungal pathogen Candida albicans must adapt to various host microenvironments during the colonization of host mucosal surfaces. The availability and management of nutrients, including carbohydrates, at diverse biological sites helps C. albicans grow, survive, and adapt to changing environmental conditions. It is widely recognized that in C. albicans and other yeasts, such as Saccharomyces cerevisiae (S. cerevisiae), glycolysis plays vital roles in carbon and energy accumulation, aerobic and anaerobic metabolism, as well as the fundamental maintenance of the carbohydrates glycogen, glucan, mannan, and chitin that are found in cell walls (1–4).

**FIG 1:**
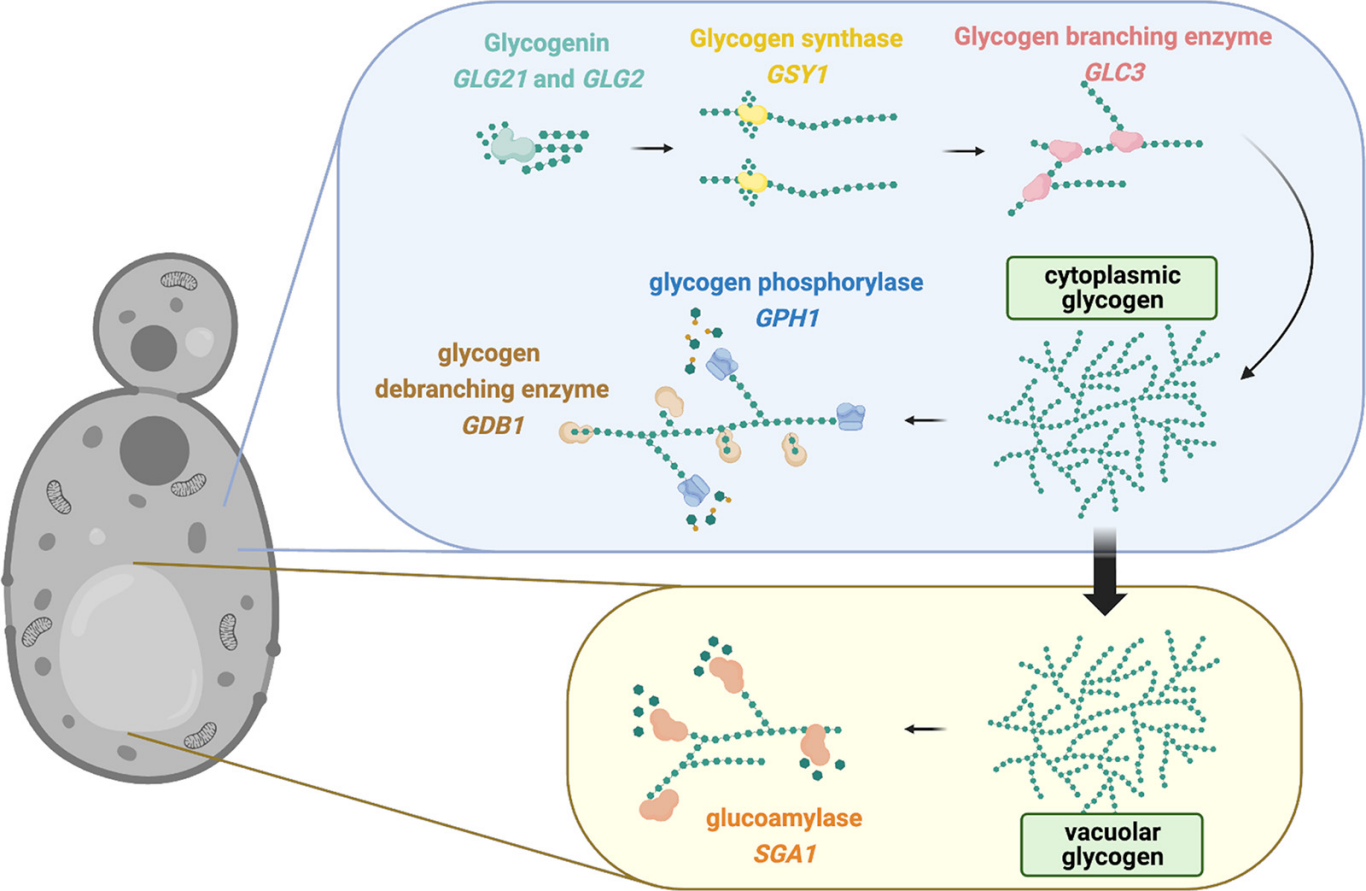
Inferred glycogen synthesis and catabolism pathways in C. albicans, based on orthologous functions in S. cerevisiae. Glycogen synthesis is initiated by a glucosyltransferase (otherwise referred to as glycogenin and encoded by *GLG2* or *GLG21*) that converts UDP-α-glucose into short α-1,4-linked glucosyl chains that are covalently attached to form a nucleate core. The glycogen synthase (encoded by *GSY1*) extends short α-1,4-linked glucosyl primers into elongated chains. The extensive branching of the mature glycogen molecule is achieved via the activity of the glycogen branching enzyme (encoded by *GLC3*) to introduce α-1,6-linkages. Glycogen is catabolized in a reverse fashion by the glycogen debranching enzyme (encoded by *GDB1*) to remove α-1,6-linkages, and this is followed by the activity of the glycogen phosphorylase (encoded by *GPH1*) to generate α-glucose-1-phosphate monomers from α-1,4-linked glucose via the addition of a phosphate. α-glucose-1-phosphate can be further metabolized to enter glycolysis. Yeast can also store glycogen in the vacuole, where glucoamylase (encoded by *SGA1*) can rapidly degrade glycogen to α-glucose-1-phosphate during sporulation. However, this pathway is less understood than that of conventional *GDB1*-*GPH1*-mediated glycogen catabolism. Orthologous sequences were identified by using the Candida Genome Database. The figure was created using BioRender.

Glycogen biosynthesis and catabolism are important metabolic strategies by which C. albicans and other yeasts cope with starvation, and they have been well-studied in the model yeast species S. cerevisiae (5, 6). As depicted in Fig. 1, glycogen synthesis begins via the conversion of UDP-glucose into short α-1,4-glucosyl oligosaccharide primers by a glucosyltransferase (encoded by *GLG21* or *GLG2*), which is more commonly referred to as glycogenin. These primers are covalently attached to glycogenin to form a nucleation site for the next step of glycogen synthesis. The elongation of linearized glycogen through α-1,4 linkages is accomplished by glycogen synthase (encoded by *GSY1*) and is followed by the introduction of α-1,6 branch points via the branching enzyme (encoded by *GLC3*) to build the branched polymer that is referred to as glycogen (5, 7–10). Intracellular glycogen can ultimately be stored in the cytoplasm and vacuole. Thus, two catabolism pathways exist. The cytoplasmic degradation of glycogen occurs via glycogen phosphorylation from the nonreducing ends of α-1,4 linked chains (driven by a glycogen phosphorylase that is encoded by *GPH1*), and the debranching enzyme (encoded by *GDB1*) hydrolyzes α-1,6 linked glucose residues to produce glucose-1-phosphate. Second, glycogen can be directly hydrolyzed to free glucose by vacuolar α-glucoamylase (encoded by *SGA1*) (5, 11–13). Glycogen synthesis and storage in S. cerevisiae is tightly coordinated via transcriptional circuits, including the Snf1/Pka pathway, to control the glycogen synthase abundance and activation states of both the synthase and phosphorylase via cyclin-dependent protein kinases (14–18). However, it has not yet been determined whether the homologues that are predicted to drive glycogen synthesis and catabolism function similarly in the opportunistic pathogen C. albicans.

Vulvovaginal candidiasis (VVC) is an infection of the vulva and/or vagina that is caused by *Candida* spp., with 85% to 95% of the cases being caused by C. albicans (19–21). According to the Centers for Disease Control (CDC) and other reports, VVC presents a significant economic impact, resulting in an estimated $368 million in medical costs in the United States per year (22, 23). It is believed that the development of VVC requires the prior asymptomatic colonization of the vaginal mucosa and is associated with vaginal dysbiosis that results from behavioral or other host-related factors, such as pregnancy, hormone replacement therapy, estrogen-containing oral contraceptives, or antibiotic use (24–26). In fact, increased estrogen activity (e.g., as observed during the ovulatory phase of the menstrual cycle) promotes epithelial glycogen accumulation, and free glycogen may exceed glucose levels by approximately 10-fold in the vagina (27, 28). A prior report also suggests that C. albicans is uniquely suited to assimilate glycogen during *in vitro* growth (29). Thus, it is reasonable to hypothesize that high glycogen levels in the vaginal environment may positively impact C. albicans survival and its capacity to drive the onset of VVC. Aside from mucosal disease, C. albicans can also cause cases of life-threatening invasive mycosis, in which it may inhabit body organs and must adapt to unique nutrient availabilities, including possibly glycogen (30).

Given these points and the lack of studies regarding the potential connection between glycogen and candidiasis, we wished to conclusively investigate the glycogen metabolism pathways of C. albicans as well as their impacts on fitness at the vaginal mucosa and virulence during invasive diseases. Using a collection of reference and vaginal clinical isolates, we first determined that C. albicans growth is poorly supported when glycogen is supplied as the sole carbon source. Second, the construction of a panel of deletion mutants validated glycogen phosphorylase (*GPH1*) and debranching enzymes (*GDB1*) as the major contributors to glycogen catabolism, with vacuolar glucoamylase (*SGA1*) playing a minor, compensatory role. Additional experiments revealed that *GSY1* and *GLC3* play key roles in glycogen synthesis. Competitive survival assays confirmed that both *gsy1*Δ/Δ and *gph1*Δ/Δ *sga1*Δ/Δ mutants exhibited long-term survival defects *in vitro* under starvation conditions and *in vivo* during vaginal colonization. Moreover, the median survival of mice that were intravenously challenged with the *gph1*Δ/Δ *sga1*Δ/Δ mutant was significantly higher than that of the WT strain or of a *gph1*Δ/*Δsga1*Δ/Δ+*GPH1* strain. Collectively, these results provide strong evidence that glycogen metabolism provides a fitness advantage at the vaginal mucosa, and an inability to catabolize glycogen impacts virulence during disseminated infections.

## RESULTS

### Extracellular glycogen is a poor substrate for C. albicans growth.

**FIG 2:**
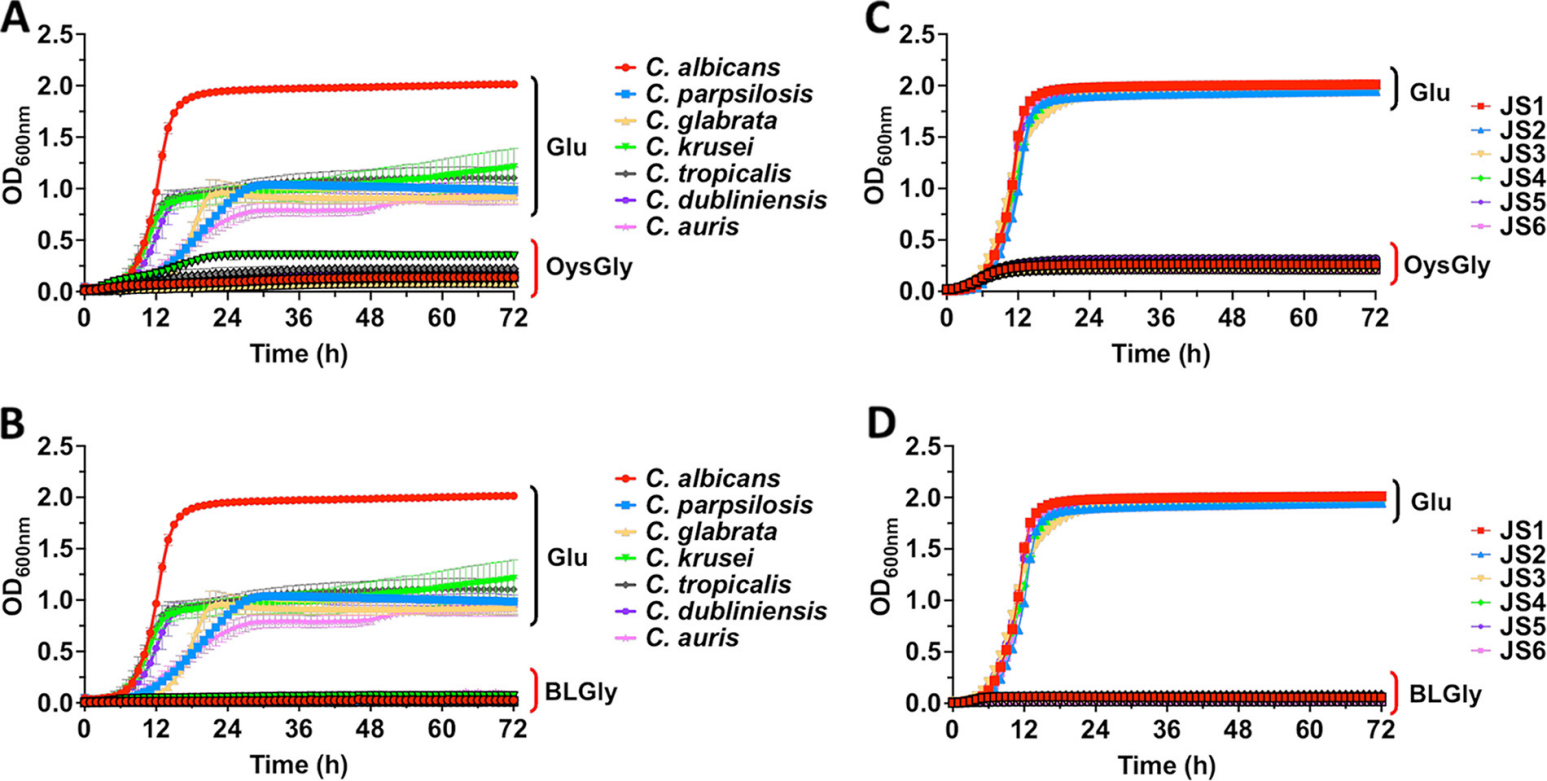
Extracellular glycogen as a sole carbon source is poorly utilized by the *Candida* species. C. albicans, C. dubliniensis, C. krusei, C. glabrata, C. tropicalis, C. parapsilosis, and C. auris were grown in YNB containing 0.5% glucose or 0.5% (A) oyster glycogen (OysGly) or (B) bovine liver glycogen (BLGly), and the OD_600_ value was recorded. Similar experiments were performed with the C. albicans vaginal clinical isolates JS1, JS2, JS3, JS4, JS5, and JS6 in 0.5% glucose or 0.5% (C) OysGly or (D) BLGly. All experiments were repeated in triplicate, and the data are presented as the mean ± SEM.

A prior study demonstrated that C. albicans, but not non-*albicans Candida* species, was capable of assimilating extracellular glycogen *in vitro* (29). Given that glycogen levels can become relatively high in the vaginal epithelium, it was reasonable to speculate that growth on this carbon source may explain the high incidence of C. albicans that is observed during VVC. However, when using YNB medium with oyster glycogen as the sole carbon source, poor growth was observed in all seven *Candida* species utilized (C. albicans, C. dubliniensis, C. krusei, C. glabrata, C. tropicalis, C. parapsilosis, and C. auris), compared to YNB medium supplemented with glucose (Fig. 2A). Similar results were observed when bovine liver glycogen was used as the sole carbon source (Fig. 2B). The observed patterns indicated that extracellular glycogen was not an optimal extracellular carbohydrate source for supporting the growth of the *Candida* species used in this study. In order to rule out potential interstrain heterogeneity exhibited by C. albicans, additional vaginal clinical isolates (JS1 to JS6) were used in a similar growth assay. There was no significant difference in the growth patterns between the various clinical isolates, yet all grew well in medium supplemented with glucose but poorly with oyster or bovine liver glycogen (Fig. 2C and D). Our findings suggest that environmental glycogen acquisition by the *Candida* species is limited and that C. albicans showed no exclusive advantage in utilizing extracellular glycogen as a carbon source.

### Functional characterization of genes involved in glycogen metabolism.

The glycogen metabolism pathways have been well-studied in S. cerevisiae, but they have not been extensively characterized in C. albicans. In order to investigate the impact of glycogen metabolism on the survival and fitness of C. albicans, we employed genetic manipulation approaches to validate genes that contribute to glycogen metabolism. For each individual C. albicans gene that is homologous with those involved in glycogen synthesis (*GLG1*, *GLG2*, *GSY1*, *GLC3*) and catabolism (*GPH1*, *GDB1*, *SGA1*) in S. cerevisiae (Fig. 1), we constructed knockout mutants and revertants. Importantly, the mutant and revertant strains grew and filamented similar to the wild-type under standard laboratory conditions in YNB and buffered RPMI medium (Fig. S1A and B). Moreover, none of the mutants that were tested exhibited susceptibility to peroxide, ionic, cell wall, osmolarity, metal ion, temperature, or pH stress, nor did their growth differ on the alternative carbon sources, namely, glycerol and ethanol (Fig. S2).

10.1128/mbio.00046-23.4 FIG S1 Glycogen metabolism mutant and revertant strains do not display general growth or filamentation defects under standard laboratory conditions. (A) WT, glycogen synthesis mutants (*glg2Δ/Δ*, *glg21Δ/Δ, gsy1Δ/Δ*, *glc3Δ/Δ*) and their revertants (*glg2Δ/Δ*+*GLG2*, *glg21Δ/Δ*+*GLG21*, g*sy1Δ/Δ*+*GSY1*, *glc3Δ/Δ*+*GLC3*), or glycogen catabolism mutants (*gdb1Δ/Δ*, *gph1Δ/Δ*, *sga1Δ/Δ*, *gph1Δ/Δ sga1Δ/Δ*) and their revertants (*gdb1Δ/Δ*+*GDB1*, *gph1Δ/Δ*+*GPH1*, *sga1Δ/Δ*+*SGA1*, *gph1Δ/Δ sga1Δ/Δ*+*GPH1*, *gph1Δ/Δ sga1Δ/Δ*+*SGA1*) were inoculated in YNB medium containing 0.5% glucose, and their growth was monitored for 48 h at 30°C with shaking by measuring the OD_600_ value. (B) These same strains were inoculated in MOPS-buffered RPMI-1640 (pH 7.0) and grown for 4 h at 37°C with shaking. Filamentation was digitally captured via light microscopy (scale bar = 50 μm). Download FIG S1, TIF file, 2.5 MB. Copyright © 2023 Miao et al. 2023 Miao et al. https://creativecommons.org/licenses/by/4.0/ This content is distributed under the terms of the Creative Commons Attribution 4.0 International license.

10.1128/mbio.00046-23.5 FIG S2 C. albicans glycogen mutants show no sensitivity to osmotic, oxidative, pH, temperature, or cell wall stress or growth differences on alternative carbon sources, compared to the wild-type strain. C. albicans overnight cultures were washed twice and adjusted to 2 × 10^7^ cells/mL. The indicated serial dilutions were spotted (5 μL) onto YP agar plates containing dextrose, glycerol, or ethanol (as noted) either alone or containing the specified stressors. Unless noted otherwise, the plates were incubated at 30°C for 48 h, prior to digital image acquisition. The data are representative of two independent repeats. Download FIG S2, TIF file, 3.1 MB. Copyright © 2023 Miao et al. 2023 Miao et al. https://creativecommons.org/licenses/by/4.0/ This content is distributed under the terms of the Creative Commons Attribution 4.0 International license.

**FIG 3:**
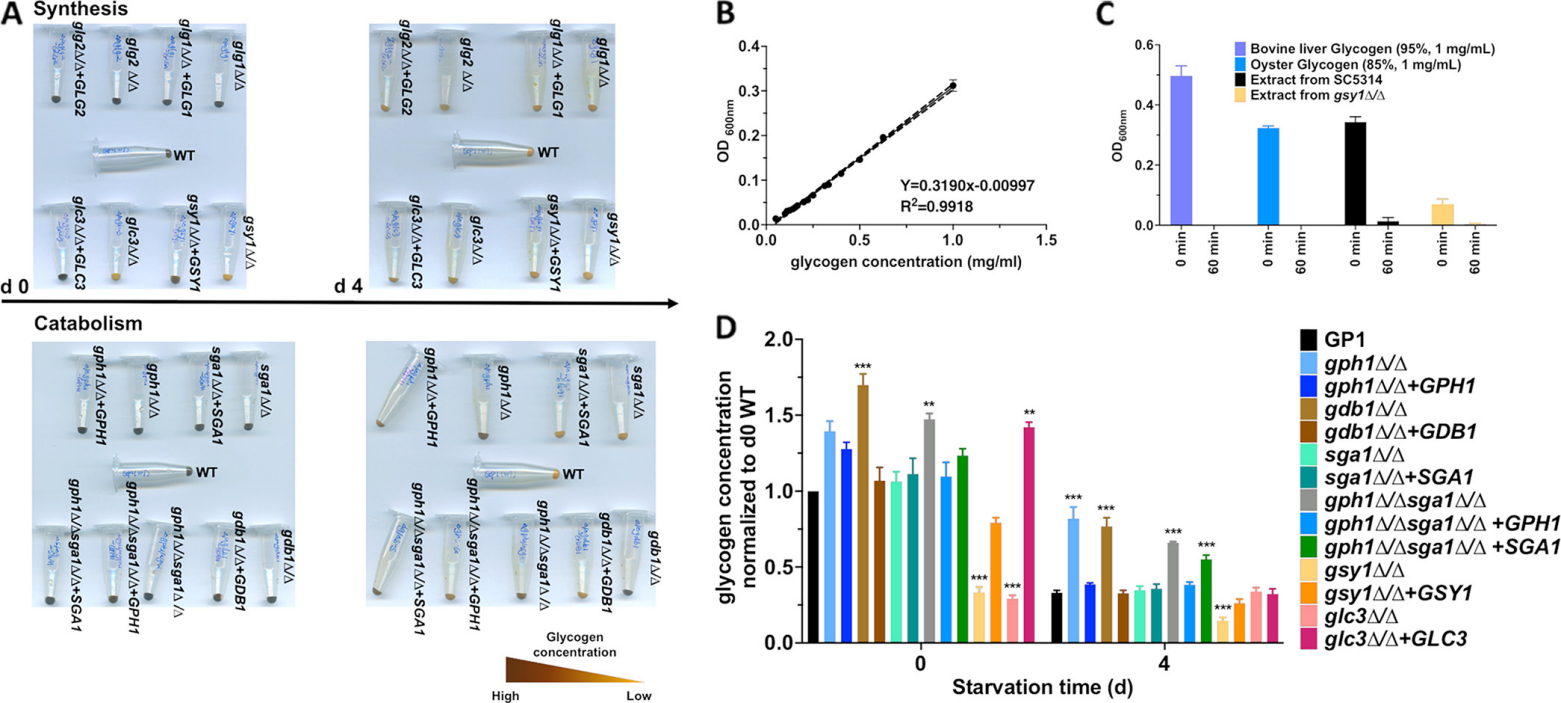
The deletion of predicted glycogen metabolism genes confirm function in C. albicans. (A) C. albicans glycogen synthesis mutants (*glg2Δ/Δ*, *gsy1Δ/Δ*, *glc3Δ/Δ*) and revertants (*glg2Δ/Δ*+*GLG2*, *gsy1Δ/Δ*+*GSY1*, *glc3Δ/Δ*+*GLC3*) were grown overnight in YNB medium, and either cell pellets were stained immediately with iodine solution or cells were starved for 4 days in YNB media lacking a carbon source and then stained. Similar experiments were performed using glycogen catabolism mutants (*gdb1Δ/Δ*, *gph1Δ/Δ*, *sga1Δ/Δ*, *gph1Δ/Δsga1Δ/Δ*) or revertants (*gdb1Δ/Δ*+*GDB1*, *gph1Δ/Δ*+*GPH1*, *sga1Δ/Δ*+*SGA1*, *gph1Δ/Δsga1Δ/Δ*+*GPH1*, *gph1Δ/Δsga1*+*SGA1*). Images were captured on a digital scanner and are representative of 3 independent experiments. (B) A standard curve was generated by iodine staining a series of oyster glycogen dilutions and plotting the OD_600_ nm values. Curves were generated in triplicate to demonstrate reproducibility, and the data are depicted as the mean ± SEM. (C) Extracted material from WT or *gsy1Δ/Δ* strains or 1 mg of resuspended commercial oyster or bovine liver glycogen was stained with iodine solution pre- (0 min) and post- (60 min) enzymatic treatment with glucoamylase. The data are presented as the mean ± SEM. (D) Glycogen was extracted from the above-described strains, and the precipitate was resuspended and stained with iodine solution. The data were interpolated from a standard curve of oyster glycogen, were normalized to the WT, and are depicted as the mean + SEM. A one-way ANOVA with Dunnett’s post-test was used for the statistical analyses. *, *P* < 0.05; **, *P* < 0.01; ***, *P* < 0.001.

Both qualitative and quantitative iodine staining approaches were used to assess glycogen levels at baseline and following starvation (31). Strains were grown overnight in YNB supplemented with glucose (day 0) and were transitioned to YNB lacking a carbon source to induce starvation, where they were incubated for 4 days (day 4). A notable glycogen synthesis defect was observed in the *gsy1Δ/Δ* and *glc3Δ/Δ* strains, compared to the WT strain GP-1 at day 0, as indicated by a lack of dark brown coloration following iodine staining (Fig. 3A). These phenotypes were reversed by gene complementation. No defect was observed for the *glg2*Δ/Δ or *glg21*Δ/Δ mutants, as these genes likely have compensatory functions, given their similar predicted roles. Glycogen catabolism defects were observed in the *gph1*Δ/Δ, *gdb1*Δ/Δ, *gph1*Δ/Δ *sga1*Δ/Δ, and *gph1Δ/Δ sga1*Δ/Δ*+SGA1* strains after starvation (day 4), whereas the revertant and *gph1Δ/Δ sga1*Δ/Δ+*GPH1* strains regained their capacities to utilize glycogen, similar to the WT (Fig. 3A). A more accurate quantitative approach was utilized, in which the glycogen fraction was extracted from C. albicans, stained with iodine, and compared to a standard curve that was generated using oyster glycogen (Fig. 3B) (12, 31, 32). In order to verify whether iodine staining was sensitive to glycogen, extracts from the WT and *gsy1*Δ/Δ strains, as well as aliquots of commercial bovine liver and oyster glycogen, were stained before and after enzymatic digestion with α-amylase (1). The iodine signal was almost completely ablated post-treatment, suggesting that the extraction procedure does indeed precipitate glycogen and that this assay accurately reflects the cellular glycogen content (Fig. 3C). Generally, the results paralleled those of the qualitative assay, with the exception that the *gph1*Δ/Δ+*GPH1* strain appeared to demonstrate elevated glycogen levels at baseline (Fig. 3D). Collectively, these data indicated that the glycogen synthesis pathways in C. albicans and S. cerevisiae are generally conserved and are controlled by similar genes. Unlike in S. cerevisiae, there does not appear to be a functionally redundant glycogen synthase paralog in C. albicans (33). Similarly, the main glycogen catabolism pathway of C. albicans was found to be mediated by glycogen phosphorylase (*GPH1*) and glycogen debranching (*GDB1*) enzymes, and this was confirmed by elevated glycogen levels being detected in the *gph1Δ/Δ sga1*Δ/Δ*+SGA1* strain, compared to the *gph1Δ/Δ sga1*Δ/Δ*+GPH1* strain, suggesting that *SGA1* cannot compensate for the loss of *GPH1* or *GDB1* under these specific *in vitro* conditions. Thus, we conclude that the critical genes that contribute to C. albicans intracellular glycogen synthesis are *GSY1* and *GLC3*, whereas catabolism is driven largely by *GPH1* and *GDB1*.

### Glycogen metabolism contributes to the long-term survival of C. albicans *in vitro*.

**FIG 4:**
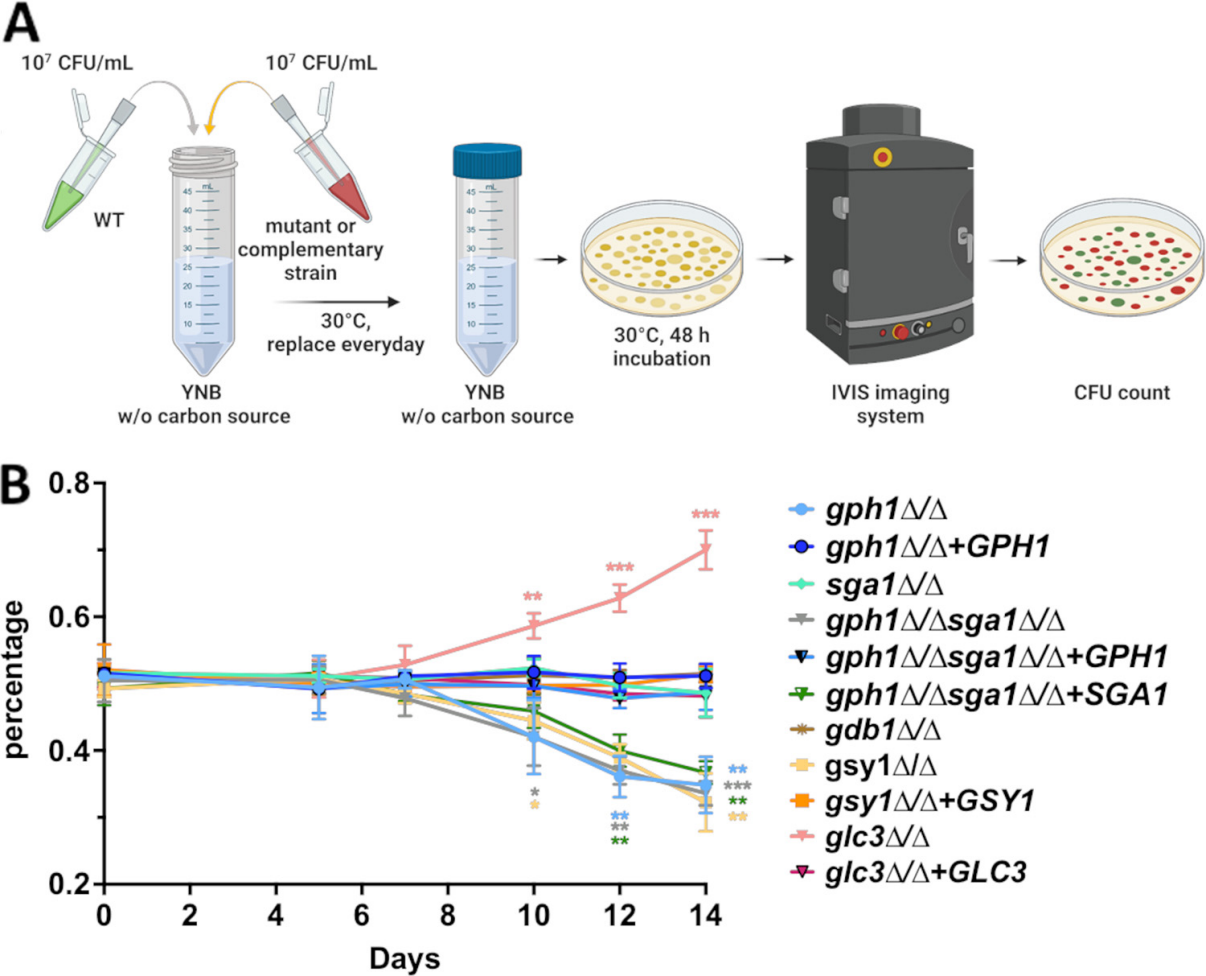
Disruption of glycogen metabolism impacts fitness during *in vitro* starvation. (A) Schematic depicting the experimental design. GFPy-tagged WT and dTomato-tagged glycogen metabolism mutant or revertant strains were mixed in equal proportions and subjected to long-term starvation in YNB lacking a carbon source. At days 0, 5, 7, 10, 12, and 14 post-inoculation, aliquots were removed, density-adjusted, and plated onto YPD medium. Fluorescence images of the agar plates were captured using an IVIS Spectrum imager. (B) Fluorescent CFU were enumerated from the images, and those exhibiting red fluorescence were reported as a percentage of the total. The data are depicted as the mean ± SEM. A multiple *t* test was used to compare each mutant and revertant percentage at each time point. *, *P* < 0.05; **, *P* < 0.01; ***, *P* < 0.001.

We have demonstrated that extracellular glycogen, as a sole carbon source, does not robustly support C. albicans proliferation. However, C. albicans may exist indefinitely as a member of the normal vaginal microbiota, prior to driving a symptomatic infection and intracellular glycogen content. Also, its metabolism may play a role in fungal fitness and in the colonization of the dynamic vaginal environment. Thus, we used an *in vitro* competitive growth assay under starvation conditions with fluorescently-tagged WT and mutant or revertant strains to indirectly test this hypothesis (Fig. 4A). The *gsy1*Δ/Δ, *gph1*Δ/Δ, *gph1Δ/Δ sga1*Δ/Δ, and *gph1Δ/Δ sga1*Δ/Δ+*SGA1* strains demonstrated significantly reduced competitive fitness, compared to the wild-type strain, and this survival defect was largely rescued via gene complementation (Fig. 4B). One exception was that the activity of *SGA1* was not sufficient to compensate for the loss of *GPH1* in the double mutant under long-term starvation conditions. This further confirms a dominant role for *GPH1* in the driving of glycogen catabolism. Surprisingly, the *gdb1*Δ/Δ mutant did not exhibit a competitive fitness disadvantage, despite its confirmed role in glycogen catabolism. Also, unexpectedly, the *glc3*Δ/Δ mutant was more fit than was the wild-type under these conditions (Fig. 4B).

### The disruption of glycogen metabolism impacts C. albicans fitness and virulence.

**FIG 5:**
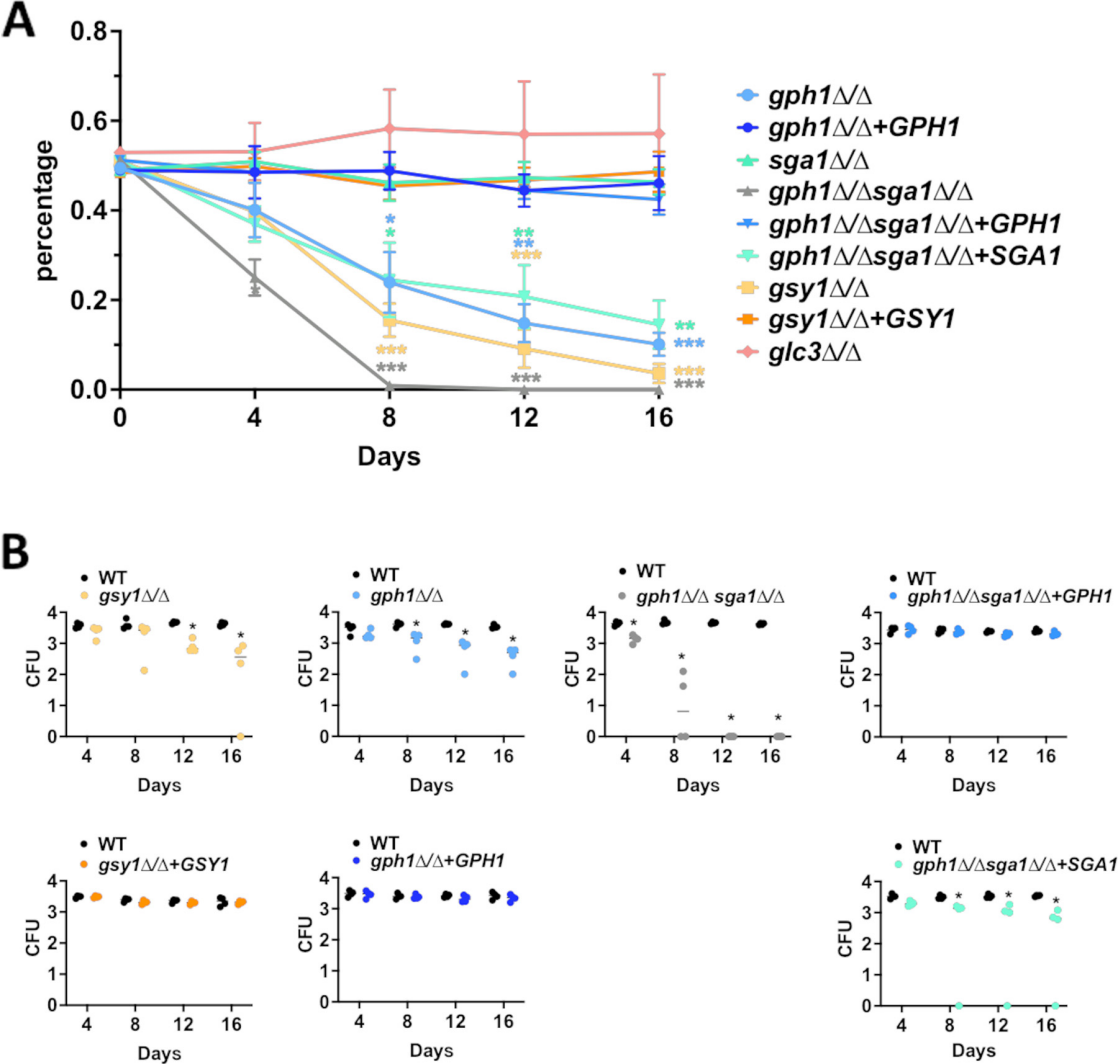
Glycogen synthase, glycogen phosphorylase, and glucoamylase mutants are less competitively fit during vaginal colonization. (A) Groups of mice (*n* = 4) were inoculated with mixed cultures of GFPy-tagged WT or dTomato-tagged glycogen metabolism mutant or revertant strains. At day 0 (inoculum), 4, 8, 12, and 16 (lavage) samples were plated onto YPD agar plates containing 50 μg/mL chloramphenicol, and fluorescent CFU were enumerated via IVIS imaging. Strains are depicted in the figure legend. The data are expressed as the percentage of red fluorescent colonies that were recovered and are depicted as the mean ± SEM. A multiple *t* test was used to compare each mutant and revertant percentage at each time point. *, *P* < 0.05; **, *P* < 0.01; ***, *P* < 0.001. (B) CFU obtained from vaginal lavage samples that were obtained at days 4, 8, 12, and 16 were enumerated. The line represents the median. A multiple *t* test was used to compare each mutant and revertant percentage at each time point. *, *P* < 0.05.

**FIG 6:**
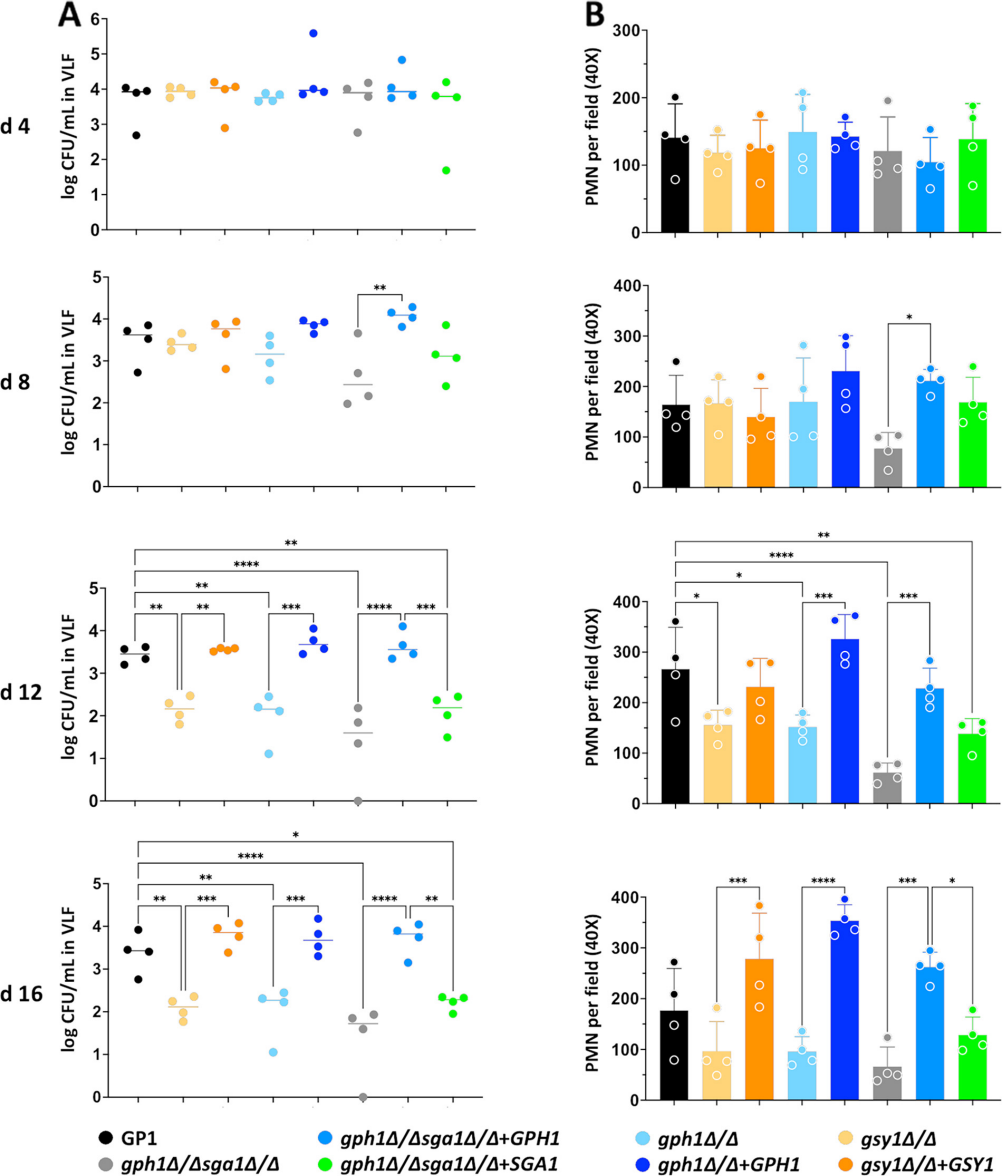
Glycogen synthase, glycogen phosphorylase, and glucoamylase mutants exhibit reduced colonization and immunopathology during murine vaginitis. Vaginal lavage fluids (VLF) obtained from mice (*n* = 4 per group) were analyzed at days 4, 8, 12, and 16 post-inoculation for (A) fungal burden (medians) and (B) PMN recruitment, as determined via microscopy (mean ± SD). The statistical testing of significance was achieved by using a one-way ANOVA with the Kruskal-Wallis (CFU data) or with Tukey’s (PMN data) post-test, and statistical significance is denoted as follows: *, *P* < 0.05; **, *P* < 0.01; ***, *P* < 0.001.

In order to understand whether glycogen metabolism impacts C. albicans fitness at the vaginal mucosa, we employed a standard murine model of VVC that is amenable to the longitudinal analysis of fungal colonization (34–36). Both the survival rate and the absolute quantitative counts were analyzed to determine whether C. albicans competitive fitness at the vaginal mucosa could be impacted by disrupted glycogen metabolism pathways. Similar to *in vitro* findings, the *gsy1*Δ/Δ, *gph1*Δ/Δ, *gph1Δ/Δ sga1*Δ/Δ, and *gph1Δ/Δ sga1*Δ/Δ+*SGA1* strains were less fit *in vivo*, compared to the wild-type strain (Fig. 5A and B). The remaining strains colonized mice throughout the infectious time course in a manner that was similar to that of the wild-type (Fig. S3). The *glc3*Δ/Δ mutant exhibited a slightly increased survival trend *in vivo* that was similar to the *in vitro* findings, but it was not significant. The *gph1*Δ/Δ *sga1*Δ/Δ had a notable, severe competitive survival defect with no detectable CFU being recovered by day 12 postinoculation. Interestingly, unlike the *in vitro* findings, the reversion of *SGA1* in this mutant somewhat rescued the survival defect, similar to that observed with the complementation with *GPH1* (Fig. 5A and B). As neutrophil influx to the vaginal lumen is a hallmark of vaginitis immunopathology, single-strain infections were performed using *gsy1*Δ/Δ, *gph1*Δ/Δ, or *gph1*Δ/Δ *sga1*Δ/Δ mutants, and their respective complemented strains, fungal burden, and neutrophils were monitored longitudinally (35). The fungal burden was similar among all strains up to day 4 postinoculation, but the mutants were generally less fit than were the revertants or the WT at later time points (Fig. 6A). The neutrophil levels were similar among all strains at day 4, but they decreased concomitantly with the fungal burdens during infections with mutant strains (Fig. 6B). As all strains formed hyphae at the vaginal mucosa, it was unsurprising that the neutrophil levels were initially similar, given the importance of the yeast-to-hypha switch in driving immunopathogenesis (Fig. S4). Collectively, the data demonstrate that the genetic deletion of glycogen synthase, glycogen phosphorylase, and/or α-glucoamylase impairs the fitness of C. albicans at the vaginal mucosa, independent of filamentation.

10.1128/mbio.00046-23.6 FIG S3 The losses of glucoamylase and the glycogen branching enzyme have no impact on vaginal colonization. Groups of mice (*n* = 4) were inoculated with 1:1 mixed cultures of GFPy-tagged WT or dTomato-tagged glycogen metabolism mutant or revertant strains. At days 4, 8, 12, and 16, vaginal lavage samples were plated onto YPD agar plates containing 50 μg/mL chloramphenicol and fluorescent CFUs that were enumerated via IVIS imaging. The strains are depicted in the figure legend. The line represents the median. A multiple *t* test was used to compare each mutant and revertant percentage at each time point. *, *P* < 0.05. Download FIG S3, TIF file, 0.2 MB. Copyright © 2023 Miao et al. 2023 Miao et al. https://creativecommons.org/licenses/by/4.0/ This content is distributed under the terms of the Creative Commons Attribution 4.0 International license.

10.1128/mbio.00046-23.7 FIG S4 The loss of glycogen synthase, glycogen phosphorylase, and/or glucoamylase does impact neutrophil influx or filamentation during initial vaginal colonization. VLF were obtained at day 4 postinoculation and stained using the Papanicolaou technique. Images were digitally captured (scale bar = 100 μm). The red arrows depict hyphae. The small, round, blue cells are neutrophils. The data are representative of 5 fields of view from each mouse group (*n* = 4). Download FIG S4, TIF file, 5.6 MB. Copyright © 2023 Miao et al. 2023 Miao et al. https://creativecommons.org/licenses/by/4.0/ This content is distributed under the terms of the Creative Commons Attribution 4.0 International license.

**FIG 7:**
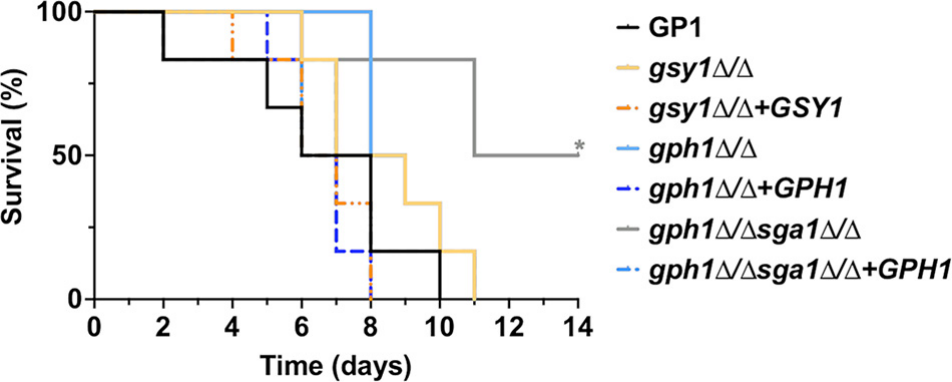
Disrupted glycogen catabolism in C. albicans reduces lethality during invasive candidiasis. Mice were intravenously inoculated with glycogen synthesis (*gsy1Δ/Δ* and *gsy1Δ/Δ* +*GSY1*) or catabolism (*gph1Δ/Δ* and *gph1Δ/Δ* +*GPH1* or *gph1Δ/Δ sga1Δ/Δ* and *gph1Δ/Δ sga1Δ/Δ* +*GPH1*) mutant or revertant strains and were assessed for survival for up to 14 days postinfection. Kaplan-Meier curves were assessed for statistical significance via a log rank test. *, *P* < 0.05.

We also determined whether glycogen metabolism contributes to virulence by using a model of invasive systemic candidiasis. Mice were intravenously challenged with strains demonstrating impacted fitness in the VVC model and were survival monitored (Fig. 7). Mice challenged with the *gph1*Δ/Δ *sga1*Δ/Δ mutant demonstrated significantly increased survival, compared to those challenged with the WT or with the *gph1*Δ/Δ*sga1*Δ/Δ*+GPH1* revertant. Whereas the competitive fitness was decreased for the *gsy1*Δ/Δ and *gph1*Δ/Δ strains in the vaginal model, the median survival was not altered in the animals that were intravenously challenged with these strains, indicating that glycogen metabolism possibly impacts the biology of C. albicans in a site-specific manner.

## DISCUSSION

Most microorganisms, including bacteria, parasites, and fungi, accumulate carbon and energy reserves to cope with starvation conditions that are temporarily present in the environment (5, 6, 37). Glycogen metabolism constitutes a key strategy that regulates systemic carbon or energy allocation for such purposes. Major advantages in using glycogen as a carbohydrate reservoir is that this macromolecule has little effect on the internal osmotic pressure of the cell, and the extensive structural branching allows for efficiently compact storage (38, 39). Numerous reports in the literature have demonstrated that the loss of glycogen accumulation via the genetic disruption of glycogen synthase across multiple microbial species, including Lactobacillus acidophilus, Escherichia coli, Streptococcus mutans, Mycobacterium tuberculosis, and S. cerevisiae, confers fitness defects, both *in vitro* and *in vivo*, in the murine gastrointestinal tract and lung (40–45). We now demonstrate that such fitness or virulence defects extend to C. albicans during both vulvovaginal and systemic candidiasis, with a more prominent role during mucosal colonization. The lack of noticeable susceptibilities to a variety of stressors *in vitro* suggests that reduced *in vivo* fitness is linked to metabolic defects that are induced by disrupted glycogen homeostasis. However, we cannot rule out the possibility that the unique or combinatorial stressors that are encountered *in vivo* may disproportionately impact glycogen mutants. In humans, a number of diseases are associated with abnormal glycogen metabolism, including glycogen storage disease (GSD), which has several presentations due to varied causal genetic predispositions, including glycogen overaccumulation in the liver (46). One approach to limiting glycogen accumulation is to target glycogen synthase with small molecule inhibitors, as it is the rate limiting step for glycogen biosynthesis (47). As the genetic disruption of glycogen accumulation (via the deletion of *GSY1*) or utilization (via the deletion of *GPH1*) significantly impaired the long-term fitness of C. albicans at the vaginal mucosa, it is intriguing to speculate whether fungal-specific small molecules could be rationally designed to deter the colonization of C. albicans at mucosal surfaces.

While glycogen metabolism has been extensively explored in S. cerevisiae, mechanistic studies in pathogenic fungi have, until now, been limited. The functions of the orthologous genes that are involved in glycogen synthesis (*GSY1*, *GLC3*) and catabolism (*GPH1*, *GDB1*, and *SGA1*) have been inferred by sequence homology, but we have now confirmed and validated these functions in C. albicans. Interestingly, Sc*GSY2* is responsible for a majority (>85%) of the glycogen synthase activity in S. cerevisiae, similar to Ca*GSY1*, with Sc*GSY1* playing a more nuanced, condition-specific role (7, 48). However, the genetic loss of total glycogen synthase activity in both yeasts is clearly not lethal (7). Unfortunately, we were unable to truly determine the impact of the genetic deletion of the glucosyltransferase glycogenin that is encoded by *GLG2*. In S. cerevisiae, *GLG2* has a paralog, namely, *GLG1* that arose from whole-genome duplication. C. albicans also possesses a homolog to *GLG2*, termed *GLG21*, which is inferred to have the same function as Sc*GLG1*. We were able to successfully generate either a *glg2*Δ/Δ or *glg21*Δ/Δ (Fig. 3A) deletion strain that showed no phenotypic difference with respect to glycogen accumulation or utilization, compared to the wild-type, suggesting a compensatory function. However, despite multiple attempts, we were unable to create a *glg2*Δ/Δ *glg21*Δ/Δ double deletion mutant, indicating that these genes possibly serve other essential roles for cellular homeostasis.

The data from this study demonstrate that various C. albicans isolates and non-*albicans Candida* species seemingly do not possess the capacity to efficiently utilize extracellular glycogen as a sole carbon source. These findings were somewhat surprising, as a report by Dennerstein and Ellis showed that all C. albicans (*n* = 30) isolates could assimilate glycogen, whereas the entire collection of NAC species that was used (*n* = 26) could not (29). However, there are important details that are absent that might explain the differences that were observed between studies, including the growth time, liquid versus solid agar growth, glycogen source and purity, and phenotypic diversity among the *Candida* isolates that were used. That said, the glycogen catabolism genes of C. albicans lack leader signal sequences that would target their secretion into the external environment. Given the relatively large sizes of glycogen particles, importers have not been described. *Lactobacillus* species that thrive in glycogen rich environments, such as the vaginal mucosa typically rely on host α-amylases that degrade the glycogen that is produced by epithelial cells to maltose and maltotriose, which support bacterial growth and subsequent lactic acid production. However, recent work has identified secreted amylopullulanases that exist in some lactobacilli that directly catabolize glycogen (49, 50). Unfortunately, a sequence alignment search of the C. albicans SC5314 genome for PulA homologs did not reveal any sequence similarity. Therefore, it is likely that C. albicans cannot use extracellular glycogen directly, but it may be able to subsist on spontaneous or host-mediated glycogen degradation products or via the release of glycogenic enzymes via autolysis. *In vitro* enzymatic or glucose isotope labeling approaches will be required to conclusively validate these hypotheses. It should also be pointed out that glycogen from different species displays heterogeneous glycogen particles with respect to size, polymerization, and branching (51–53), as demonstrated by *in vitro* enzymatic assembly assays that used branching enzymes from different species (54, 55). Such disparities in structure also alter the degradation kinetics. Thus, it is possible that C. albicans prefers or utilizes specific glycogen structures, aside from what we have tested herein. The recombinant expression and purification of C. albicans glycogen enzymes, coupled with *in vitro* degradation assays, would be desirable.

The construction of glycogen synthesis and catabolism mutants generally yielded the expected phenotypes, with respect to glycogen content and observed under basal and starvation conditions. However, we did observe hyper glycogen accumulation phenotypes in a few revertant strains, including *gph1*Δ/Δ+*GPH1* and *glc3*Δ/Δ+*GLC3*. We tested multiple transformants from separate transformations, each with the same phenotype (data not shown). One potential explanation for this is that the revertant copy was introduced at the *URA3* locus, as varied expression levels of reintegrated genes at this locus have been reported. Additionally, we chose to clone a 1 kb promoter fragment upstream of the translation start site for each revertant gene. It is possible that additional regulatory sequences were absent, compared to those found at the native loci. Moreover, a single copy of each gene was reintroduced into the mutants. So, the gene dosage could also play a minor role in the observed differences. In any case, these hyper glycogen accumulation phenotypes did not influence the validation studies for *GLC3* and *GPH1*, as the revertant strains accordingly compensated the defects in glycogen accumulation and catabolism that were observed in the mutants.

It is surprising that a survival defect was not observed in the *glc3*Δ/Δ or *gdb1*Δ/Δ strains either *in vitro* or *in vivo*. *GLC3* encodes a glycogen branching enzyme and introduces α-1,6 branch points to the otherwise α-1,4 linked linear glucose polymer to increase structural complexity, rendering it more stable for longer term storage. Iodine detects glycogen by intercalating into helical chains of >10 linked glucose polymers to from a deep brown-colored complex (31). Thus, it is possible that alternative length α-1,4-linked unbranched glucose polymers exist in the *glc3*Δ/Δ mutant that stain lesser than does fully branched glycogen but still provide readily (and perhaps more accessible) available substrate for enhanced survival under starvation conditions. In a similar but opposite fashion, *GDB1* encodes a debranching enzyme that resolves α-1,6 linkages to facilitate glycogen degradation. However, functional *GPH1* is still able to remove glucose-1-phosphate from branched glycogen but does so in a relatively inefficient manner, stalling at the α-1,6 branchpoint. Therefore, the *gdb1*Δ/Δ mutant may still be able to generate enough glucose from glycogen to survive under starvation conditions. Whereas shorter chain polysaccharides and inefficient glucose-1-phosphate generation from mature glycogen may be sufficient for survival, there is compromised capacity for C. albicans to store glucose long-term in a form that can be rapidly mobilized for glycolysis when needed.

Regarding glycogen catabolism, *SGA1* appeared to have a differential capacity to support C. albicans survival *in vitro* and *in vivo*. *SGA1* was originally described as a sporulation-specific enzyme that is responsible for the mobilization of glycogen reserves in germinating yeast spores (56, 57). More recently, *SGA1* was shown to encode a vacuolar α-glucosidase that aids glycogen degradation in this organelle during late stationary-phase growth (12). In S. cerevisiae, glycogen is delivered to the vacuole primarily via the process of autophagy (5). Our data demonstrated that restoring *SGA1* was not able to rescue the long-term survival defect of *gph1*Δ/Δ *sga1*Δ/Δ *in vitro*, whereas it was able to do so in a limited fashion *in vivo*. It is possible that alternative carbon sources exist *in vivo* that are optimal substrates for Sga1 and are not found in defined YNB medium during *in vitro* growth. It is worth noting that C. albicans mainly adopts a hyphal morphology in the murine VVC model, and it is possible that *SGA1* may play a hypha-specific role in survival. Given that normal vacuole extension is required for the yeast-to-hypha switch and for full virulence, vacuolar glycogen stores are possible important for this process or possibly contribute to hyphal survival and could possibly explain the elevated median survival during hematogenous infection with the *gph1*Δ/Δ *sga1*Δ/Δ but not the *gph1*Δ/Δ mutant. Although we did not observe any hyphal growth defects with any of the mutants, even at the vaginal mucosa, other more nutritionally complex or unique *in vivo* environments may be relevant.

Lastly, prior methods that were utilized to extract glycogen from yeast cells resulted in impure fractions containing insoluble β-(1→3)-glucan, which is a major component of the fungal cell wall that is capable of stimulating innate immune responses (58–60). Our lab has recently demonstrated that glycogen is covalently linked to β-(1→3)-β-(1→6)-glucan via the β-(1→6)-linked side chain in a number of *Candida* species, including C. albicans, C. dubliniensis, C. haemulonii, and C. auris (1). It is unclear whether glycogen is linked at the cytoplasmic or external face of the cell wall. Nonetheless, it is tempting to speculate that dysregulated glycogen metabolism may directly or indirectly affect host-pathogen interactions by altering the cell wall composition. Further studies to address this possibility are warranted and are underway in our laboratories.

### Conclusion.

Mucosal candidiasis and invasive candidiasis involve a series of complex mechanisms that underlie the interaction between C. albicans and its host. Although C. albicans exhibits flexible carbohydrate metabolism in various environments, we determined that extracellular glycogen could not be readily utilized as a substrate by C. albicans. The genetic deletion and complementation of key genes that are involved in glycogen metabolism in S. cerevisiae confirmed that *GSY1* and *GLC3*, as well as *GPH1* and *GDB1*, are essential for proper glycogen synthesis and catabolism, respectively, in C. albicans. Furthermore, using competitive *in vitro* and *in vivo* models, we have demonstrated that *gsy1*Δ/Δ, *gph1*Δ/Δ, and *gph1*Δ/Δ *sga1*Δ/Δ mutants exhibit a long-term survival defect *in vitro* under starvation conditions and a fitness defect *in vivo* during vaginal colonization. A global glycogen catabolism mutant (*gph1*Δ/Δ *sga1*Δ/Δ) is less virulent during systemic challenge, as well. These findings provide direct evidence that the C. albicans intracellular glycogen metabolism plays an important role for survival and pathogenesis at the vaginal mucosa and target organs. Overall, this is the first study to fully validate glycogen metabolism pathways in C. albicans, and the results further show that intracellular glycogen catabolism positively impacts the anatomical site-specific fitness and virulence of C. albicans in the host.

## MATERIALS AND METHODS

### Ethics statement.

The animals used in this study were housed in AALAC-approved facilities that are located in the Regional Biocontainment Laboratory (RBL) at the University of Tennessee Health Science Center (UTHSC). All of the animal work conducted in this study was approved by the UTHSC Institutional Animal Care and Use Committee under protocols 21–0265 and 19–0115. Every effort was taken to ensure that the use of animals was necessary for hypothesis testing, that the minimum number of animals required was utilized, and that steps were taken to minimize discomfort. Mice were given standard rodent chow and water *ad libitum*, and they were monitored for signs of distress, including noticeable weight loss and lethargy.

### Microorganism growth.

C. albicans and non-*albicans Candida* strains were maintained as glycerol stocks and were stored at −80°C. A small amount of stock was spread onto yeast peptone dextrose (YPD) agar and incubated at 30°C for 48 h to obtain isolated colonies. A single colony was transferred to liquid YPD and incubated at 30°C with shaking at 200 rpm for 16 h. In some experiments, C. albicans was inoculated into buffered RPMI 1640 medium (pH 7) and incubated at 37°C to assess the hyphal growth.

### Strains, RNAs, and primers.

All of the strains that were used or generated are listed in Table S1. All of the vectors that were used or generated in this study are listed in Table S2. All of the primers that were used for vector construction, strain construction, and Sanger sequencing, as well as the crRNA and gRNA sequences that were used in CRISPR-Cas9 for gene editing are listed in Table S3.

10.1128/mbio.00046-23.1 TABLE S1 List of *Candida* strains used or created in this study. Download Table S1, PDF file, 0.07 MB. Copyright © 2023 Miao et al. 2023 Miao et al. https://creativecommons.org/licenses/by/4.0/ This content is distributed under the terms of the Creative Commons Attribution 4.0 International license.

10.1128/mbio.00046-23.2 TABLE S2 List of vectors used or created in this study. Download Table S2, PDF file, 0.03 MB. Copyright © 2023 Miao et al. 2023 Miao et al. https://creativecommons.org/licenses/by/4.0/ This content is distributed under the terms of the Creative Commons Attribution 4.0 International license.

10.1128/mbio.00046-23.3 TABLE S3 List of oligonucleotides used in this study. Download Table S3, PDF file, 0.06 MB. Copyright © 2023 Miao et al. 2023 Miao et al. https://creativecommons.org/licenses/by/4.0/ This content is distributed under the terms of the Creative Commons Attribution 4.0 International license.

### Vector construction.

In order to generate strains harboring the gene of interest (GOI) promoter, entire open reading frame (ORF), and terminator, PCR products were amplified from genomic DNA that was isolated from C. albicans strain GP-1, using the primers GOI_AmpF-KpnI and GOI_AmpR-SalI (unless noted otherwise in the supplementary tables). Amplicons were digested with KpnI and SacI prior to ligation into KpnI+SacI digested pLUX (unless noted otherwise in supplementary tables) to build pLUX-GOI. In order to generate strains expressing GFPy and dTomato, plasmid DNA from pKE4-Pr*TEF1*-GFPy and pKE4-Pr*TEF1*-dTomato was amplified using the primers Pr*TEF1*_AmpF-ClaI and tADH1_AmpR-SpeI. The resulting amplicons were digested with ClaI and SpeI and were ligated into similarly digested pDUP3 to yield vector pDUP3-Pr*TEF1*-GFPy and pDUP3-Pr*TEF1*-dTomato. All of the vectors were transformed into Escherichia coli DH5α. Colonies were screened by growth on Luria-Bertani (LB) agar plates containing 100 μg/mL ampicillin. Subsequent plasmid isolation and digestion occurred with proper restriction enzymes. All plasmids were verified for the integrity of their coding sequences via Sanger sequencing (GENEWIZ, Inc.), using gene-specific primers.

### Strain construction using auxotrophic markers.

The deletion cassettes for some of the mutants that were constructed in this study were amplified via PCR with the primers GOI_DISF and GOI_DISR, using vector pRS-*ARG4*ΔSpeI or pGEM-*HIS1* as the templates. The GOIΔ/Δ *ura3*Δ/Δ deletion mutant was produced via the sequential deletion of each GOI allele, using the *HIS1* and *ARG4* markers from BWP17, transformation via the lithium acetate method, and plating on selective media, as previously described (61). The correct integration of the deletion cassettes was confirmed at each step via the PCR of genomic DNA with the primer pairs ARG4_INTF2+GOI_AMPR and ARG4_INTR2+GOI_AMPF (*ARG4* integration) or HIS1_INTR2+GOI_AMPR and HIS1_INTF2+GOI_AMPF (*HIS1* integration). The lack of an intact allele was confirmed by using the primer pair GOI_DETF+GOI_DETR. An isogenic GOIΔ/Δ mutant (EV) and reconstituted strains were produced by transforming the *ura3*Δ/Δ mutant with the following NheI-digested plasmids: pLUX (vector alone) and pLUX-GOI. The correct integration of plasmids at the *IRO1-URA3* locus was confirmed via PCR using the primer pair LUXINT_DETF+LUXINT_DETR, and restoration was verified using the primer pair GOI_DETF+GOI_DETR. In order to create the GFPy and dTomato tagged strains, pDUP3-based plasmids were digested with SfiI and were transformed into the *NEUT5* locus of relevant C. albicans strain by the lithium acetate method, as described previously (62). Single colonies were screened by plating onto a YPD agar plate containing 200 μg/mL nourseothricin. The integration at the NEUT5 locus was confirmed by using the primer pairs NEUT5L_AMPF+NAT1_INTF and NEUT5L_AMPR+tADH1_AmpR-SpeI, as well as by epifluorescence microscopy.

### Strain construction with CRISPR-Cas9 gene editing.

As we repeatedly could not generate a *gdb1Δ/Δ* strain by means of the standard techniques described above, we utilized CRISPR-Cas9 gene editing to create this strain and its revertant *Δ/Δgdb1+GDB1*. Similarly, we constructed the double-knockout strain *gph1Δ/Δ sga1Δ/Δ* (from the *gph1Δ/Δ* strain) and deleted *SGA1* or *GPH1* from single revertants to generate *gph1Δ/Δ sga1Δ/Δ+GPH1* and *gph1Δ/Δ sga1Δ/Δ+SGA1*, using CRISPR-Cas9 as described previously (63). In brief, the disruption repair templates were amplified with the primers GOI_CC9KO-F+GOI_CC9KO-R, using *SAT1*-flipper and *CaHygB*-flipper plasmids, which harbored resistance to nourseothricin and hygromycin, respectively. C. albicans cells were treated in transformation buffer (1× Tris-EDTA and 0.1 M lithium acetate [pH 7.5]) and buffer with 25 mM dithiothreitol at 30°C for 45 min and 30 min, respectively. The cells were then washed and mixed with a pre-assembled ribonucleoprotein (RNP) complex that was composed of gene-specific guide RNAs (gRNA), universal tracrRNA, 2 μg Cas9 protein, and a total of 2 μg PCR-generated repair templates (1 μg for each template). Transformation was performed using a Gene Pulser Xcell Electroporation System (Bio-RAD) with a single pulse at 1.8 kV. After recovery for 4 to 6 h at 30°C in YPD medium, the cells were plated on YPD plates containing 200 μg/mL nourseothricin (GoldBio) and 600 μg/mL hygromycin B (GoldBio). Selected colonies were then cultured overnight in yeast-peptone (YP) medium containing 2% maltose (YPM) to induce cassette excision. After selection under a lower antibiotic pressure (YPD agar plates containing 25 μg/mL nourseothricin and 75 μg/mL of hygromycin), the correct genomic integration and the excision of the resistance cassettes were confirmed via the growth phenotype on YPD, YPD + 200 μg/mL nourseothricin, and YPD + 600 μg/mL hygromycin plates as well as via PCR amplification using the primers FRT_F+GOI_AmpR, FRT_R+GOI_AmpF, and GOI_DETF+GOI_DETR. In order to generate the *gdb1Δ/Δ*+*GDB1* revertant, a repair template was PCR amplified by overlap extension PCR, which fused part of the *NEUT5* promoter and ORF region of *GDB1* and the *ADH1* terminator, which were amplified from either the GP-1 gDNA or the pDUP3-tAHD1 plasmid, as described previously (63). The Δ/Δ*gdb1* strain was edited with the CRISPR-Cas9 protocol, as described above, except that crNEUT5pDUPup crRNA was used during the RNP assembly. The transformation and genotype confirmation were then performed accordingly, as mentioned above, with the primers NAT1_INTF+NEUT5L_homology-F, PrGDB1_INTR+NEUT5L_homology-R, and GDB1_DETF+GDB1_DETR.

### Analysis of growth and stress tolerance.

*Candida* species were grown as described above, washed, and diluted to 10^5^ cells/mL in yeast nitrogen base (YNB) medium containing 0.5% oyster glycogen (Sigma-Aldrich, G8751), bovine liver glycogen (Sigma-Aldrich, G0885), or glucose. 200 μL were transferred to the wells of a microtiter plate. OD_600_ readings were captured at 60 min intervals, using a BioTek Synergy spectrophotometer with an incubation temperature of 30°C and orbital shaking at 200 rpm. The experiments were repeated in technical quadruplicate and biological triplicate. The data are expressed as the mean ± standard error of the mean (SEM). For the stress tolerance and phenotypic assays, C. albicans overnight cultures were washed twice with PBS and adjusted to 2 × 10^7^ cells/mL. Serial dilutions of cells were spotted (5 μL) onto YP agar plates containing dextrose (or other carbon sources, as indicated) under the following conditions: temperature (30°C, 37°C, or 42°C), pH (3.5 and 8.5), peroxide stress (5 mM or 25 mM H_2_O_2_), osmotic stress (1 M sorbitol), ionic stress (100 or 500 mM CaCl_2_, 1.5 M NaCl), metal ion stress (10 mM MnCl_2_), cell wall stress (25 μg/mL Congo red, 0.05% sodium dodecyl sulfate), or alternative carbon sources (3% glycerol or 3% ethanol). Unless indicated, the plates were incubated at 30°C for 48 h, prior to the acquisition of images using a Gel Doc XR Imaging System (Bio-Rad).

### Hyphal growth.

Strains were grown overnight in YNB medium with shaking at 30°C. The following day, cultures were washed with phosphate-buffered saline (PBS) via centrifugation, counted via hemocytometer, and adjusted to a final density of 5 × 10^6^ cells/mL in MOPS-buffered pH 7 RPMI 1640 medium. Cultures were returned to the incubator for 4 h of incubation at 37°C, and aliquots were then transferred to glass slides. Images were captured using a Nikon Ni-U microscope with differential images contrast (DIC), a 40× objective, and the NIS-Elements software package.

### Glycogen extraction from C. albicans.

Glycogen isolation was performed as previously described, with modification (12, 32). Aliquots of 4 × 10^8^ C. albicans cells were harvested and washed twice. The wet weights of the pellets were recorded, and the pellets were frozen in liquid nitrogen. Thawed pellets were resuspended in 200 μL 20% NaOH and boiled for 1 h with occasional vortexing. Cells were thoroughly resuspended by mixing, chilled on ice for 2 min, neutralized with 150 μL 5 M HCl, and centrifuged at 7,000 rpm for 5 min. Supernatants were transferred to chilled microtubes containing 1 mL ice-cold 95% ethanol and were incubated for 30 min at −20°C. Precipitate was collected via centrifugation and washed twice with 66% ethanol. Pellets were dried at room temperature and redissolved in PBS (pH 7.0).

### Iodine staining and standard curve.

A 40× iodine stock solution (20 mg/mL I_2_ in 40 mg/mL KI) was prepared and stored, protected from light. Cell pellets were harvested from overnight cultures in YNB and were washed twice with PBS. Pellets were then resuspended in 1 mL 1× iodine working solution and were incubated at room temperature for 10 min in the dark. Images were acquired using an EPSON digital scanner. The images are representative of 3 independent repeats. In order to quantitatively measure the cellular glycogen content, extracted glycogen pellets were dissolved in 195 μL PBS. Then, 5 μL of iodine stock solution were added. In order to generate a standard curve, oyster glycogen was dissolved in PBS, serially diluted, and similarly stained with iodine working solution. The OD_600_ was measured from both experimental and standard samples, the blank subtracted values were calculated, and a curve was generated by using a linear regression in GraphPad Prism (64). The glycogen concentration (mg/mL) was interpolated, transited to a mg/g value, based on the wet weights of pellets, as described previously, and normalized to the day 0 WT. The experiments were repeated in biological triplicate, and the data are presented as the mean ± SEM.

### Amylase treatment of glycogen and cell extracts.

As described previously (1), α-amylase (Sigma-Aldrich, catalog number A4551) was reconstituted in PBS at 0.5 mg/mL. The glycogen fractions that were extracted from SC5314 and *gsy1Δ/Δ* or aliquots of commercial bovine liver or oyster glycogen (Sigma-Aldrich) were reconstituted in PBS and treated with α-amylase or PBS. Samples were incubated at 37°C for 60 min on a rotary shaker (20 rpm). Aliquots (200 μL) were stained with iodine solution pre- and post-amylase treatment, as described above, and were then measured at OD_600_.

### *In vitro* competitive survival assay.

Overnight cultures of both the GFPy-tagged GP-1 strain and the dTomato-tagged glycogen mutants or revertants were washed, counted, and combined at a 1:1 ratio (10^7^ CFU/mL each) in YNB without glucose (pH 7.5). Each day, cells were pelleted via centrifugation, and fresh medium was added to prevent the buildup of waste products. Cultures were enumerated using a Novocyte 3000 flow cytometer, and the concentration was adjusted to approximately 10^3^ cells/mL, prior to the spreading of 100 μL aliquots on YPD plates for CFU counting on days 0, 5, 7, 10, 12 and 14. Plates were incubated at 30°C for 48 h, prior to *In Vivo* Imaging System (IVIS) imaging, which was done to capture the fluorescence. The experiments were repeated in biological triplicate. The fluorescent CFU were enumerated from the images, and the red fluorescence was expressed as a percentage of the total CFU recovered.

### Murine model of VVC.

The VVC murine model was performed as described previously, with slight modification (34–36). In short, groups of 6- to 8-week-old female C57BL/6 mice (*n* = 4) were purchased from Charles River Laboratories and were housed in isolator cages that were mounted on ventilated racks. Mice were subcutaneously administered 0.1 mg β-estradiol 17 valerate (estrogen, E2) that was dissolved in sesame oil 3 days prior to vaginal lavage or challenge with C. albicans. Thereafter, E2 was administered weekly. For the mono-infections, stationary-phase cultures of C. albicans strains were washed, counted, and then adjusted to 5 × 10^8^ cells/mL in sterile endotoxin-free PBS. For the competitive survival experiments, stationary-phase cultures of GFPy-tagged and dTomato-tagged C. albicans strains were washed, counted, and then mixed 1:1 at a total density of 5 × 10^8^ cells/mL. The mice were intravaginally inoculated with 10 μL of cell suspension, which generated an inoculum size of 5 × 10^6^ blastoconidia. The mice underwent vaginal lavage every 4 days with 100 μL of PBS until day 16 postinoculation.

### Assessment of vaginal fungal burden and immunopathology.

For the mono-infections, the recovered vaginal lavage fluids (VLF) were spiked with 100× EDTA-free protease inhibitors (cOmplete; Roche). Fresh VLF (10 μL) was smeared onto Tissue Path Superfrost Plus Gold slides (Fisher Scientific), allowed to air dry, fixed with CytoPrep spray fixative (Fisher Scientific), and stored at room temperature. The VLFs were then centrifuged, and the supernatants were transferred to new tubes and stored at −80°C. Tubes containing pellets were weighed before and after the removal of the supernatant so as to calculate the amount of PBS with which to resuspend the pellet for microbiological plating. For the assessment of the fungal burden, 50 μL aliquots were serially diluted, plated on YPD agar plates containing 50 μg/mL chloramphenicol, and subsequently incubated at 30°C for 48 h. The resulting colonies were enumerated and reported as the median. Slides containing fixed lavage fluids were stained using the Papanicolaou technique so at to enumerate the polymorphonuclear leukocytes (PMNs), as identified by their morphologies, staining appearances, and characteristic trilobed nuclei (35, 65). For each smear, the PMNs were manually counted in five nonadjacent fields via standard light microscopy, using a 40× objective. The PMN counts were averaged per field, and the values are reported as the mean PMN count per group ± the standard deviation (SD). The slides were also scanned using a Hamamatus NanoZoomer-SQ Digital slide scanner (20×) to detect hyphal growth. The fungal burdens were similarly assessed for the *in vivo* competitive survival assay, except that the fluorescence images were captured via IVIS and that the colonies were enumerated. The data are presented either as a percentage of the green or red fluorescent colonies over the total (mean ± SEM) or as raw CFU counts (median).

### IVIS imaging.

Images were captured using an *In Vivo* Imaging System (IVIS) Spectrum, following the instructions from the manufacturer. Wavelengths of λ_ex_ = 480nm/λ_em_ = 510nm and λ_ex_ = 535nm/λ_em_ = 580nm were selected for GFPy and dTomato, respectively. The experimental setup was validated via the blinded mixing of various ratios of GFPy and dTomato cultures, prior to plating and to the back-calculation of the relative percentages. The images that were retrieved from the green and red channels were saved and merged for CFU counting, using the ImageJ software package.

### Mouse model of disseminated candidiasis.

C. albicans overnight cultures were washed twice in sterile, endotoxin-free phosphate-buffered saline (PBS) and were resuspended in PBS. The cell density was determined via hemocytometer and was diluted to 7.5 × 10^6^ cells/mL. Groups of 6- to 8-week-old female CD-1 mice (*n* = 6) were inoculated with 0.1 mL of cell suspension via lateral tail vein injections. The mice were monitored three times daily for 14 days postinfection, with their weights being measured every other day. Those showing distress were euthanized. The inoculum densities were confirmed by plating dilutions of the cell suspension onto YPD agar plates and counting the colonies after 72 h.

### Figure and graphic construction.

The images were constructed in NDP.view2 (2.9.29), GraphPad Prism (9.3.1), Microsoft PowerPoint (16.67), or BioRender (biorender.com, agreement number AM24EAILXC). Any adjustments to the brightness or the contrast were applied evenly across the images. High-resolution, publication-compliant images were generated using Adobe Photoshop (23.5.0).

### Statistical analysis.

All of the data were plotted and analyzed for statistical significance using GraphPad Prism. The data were compared using a one-way analysis of variance (ANOVA), multiple unpaired *t* test, and either Dunnett’s or Tukey’s post-tests, depending on whether the data were normally distributed. The graphs are annotated to indicate the levels of the statistical significance of the results (*, *P* < 0.05; **, *P* < 0.01; ***, *P* < 0.001).

### Data availability.

The original contributions that are presented in the study are included in the article text, figures, and Supplemental Material. Further inquiries can be directed to the corresponding authors.
