# Supplementary figures and images for: Glycogen Metabolism in Candida albicans Impacts Fitness and Virulence during Vulvovaginal and Invasive Candidiasis
Source: mBio. 2023 Feb 22;14(2):e00046-23. doi: 10.1128/mbio.00046-23 (PMC10127583; doi:10.1128/mbio.00046-23)

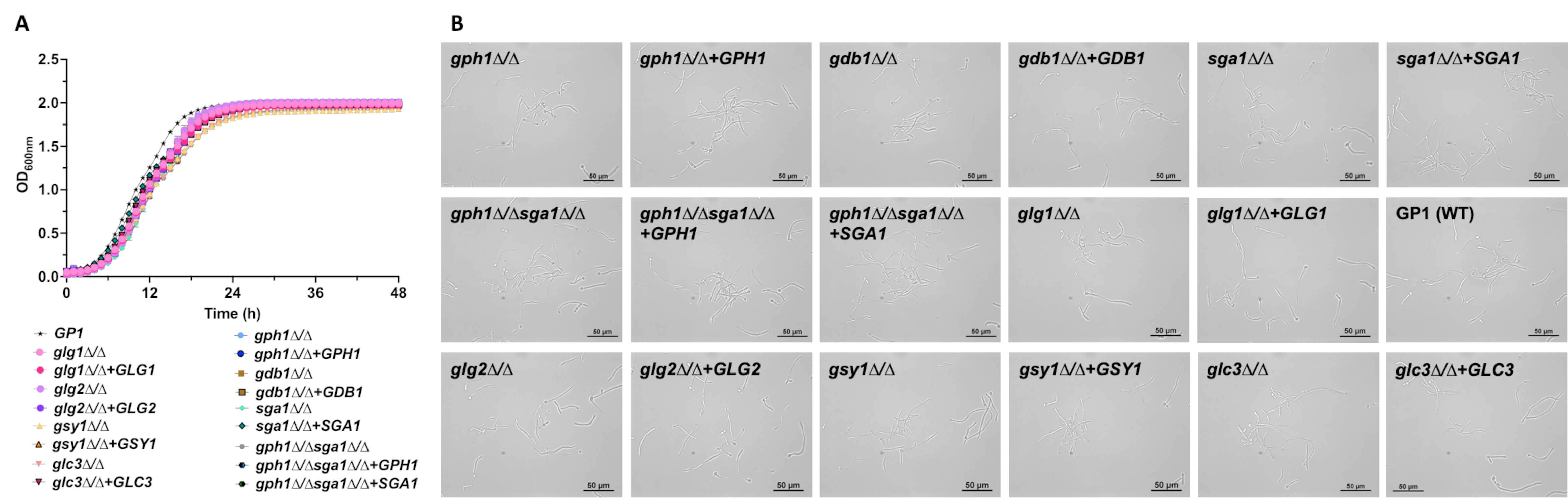

Supplement: FIG S1 [file mbio.00046-23-s0001.tif]

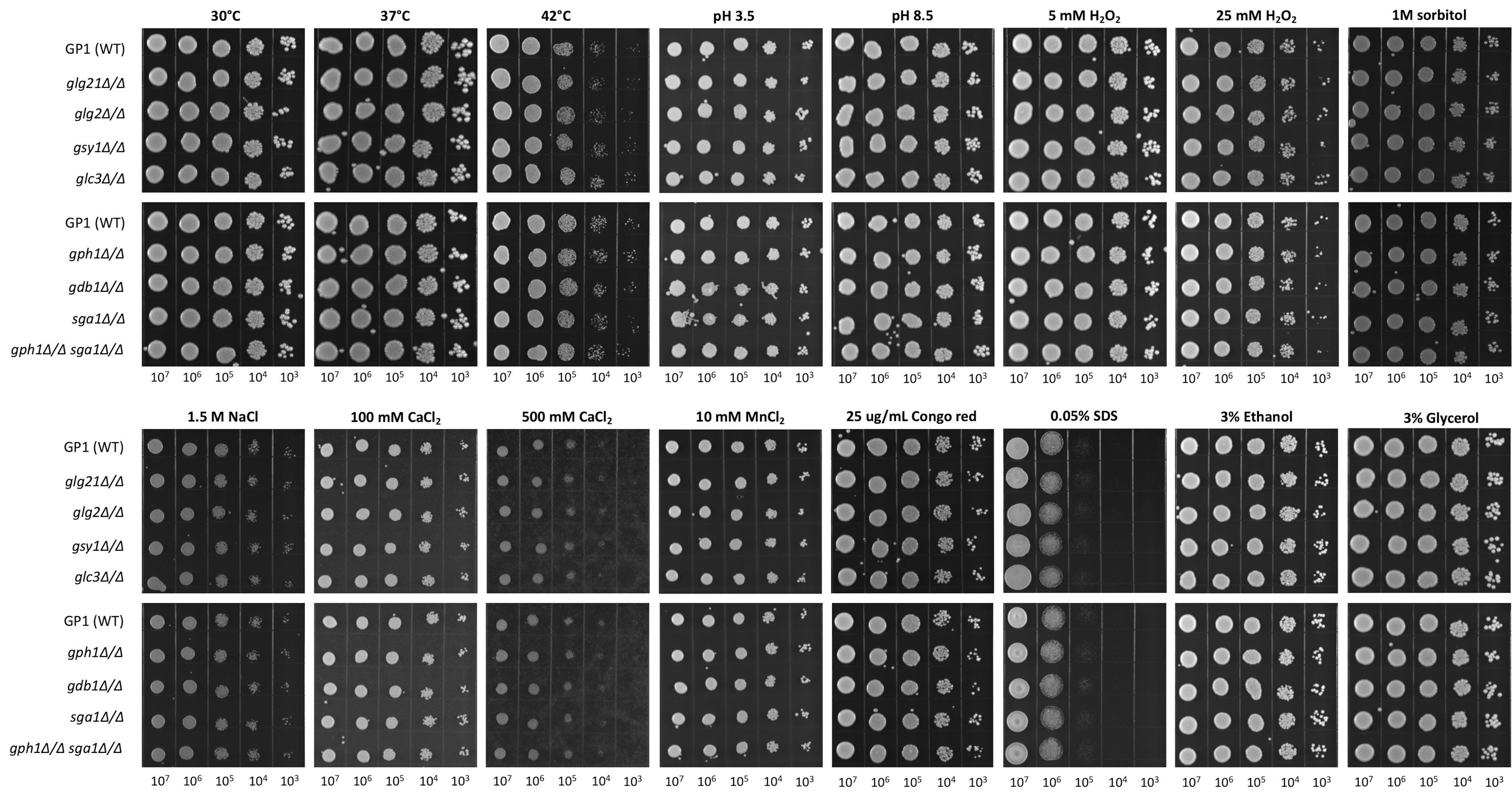

Supplement: FIG S2 [file mbio.00046-23-s0002.tif]

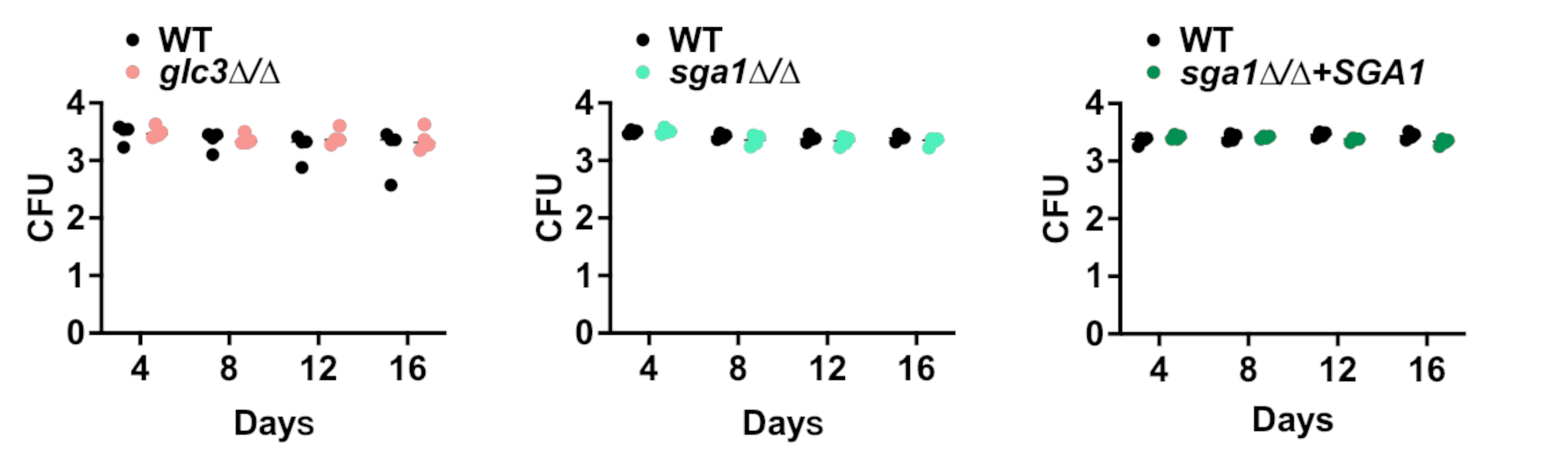

Supplement: FIG S3 [file mbio.00046-23-s0003.tif]

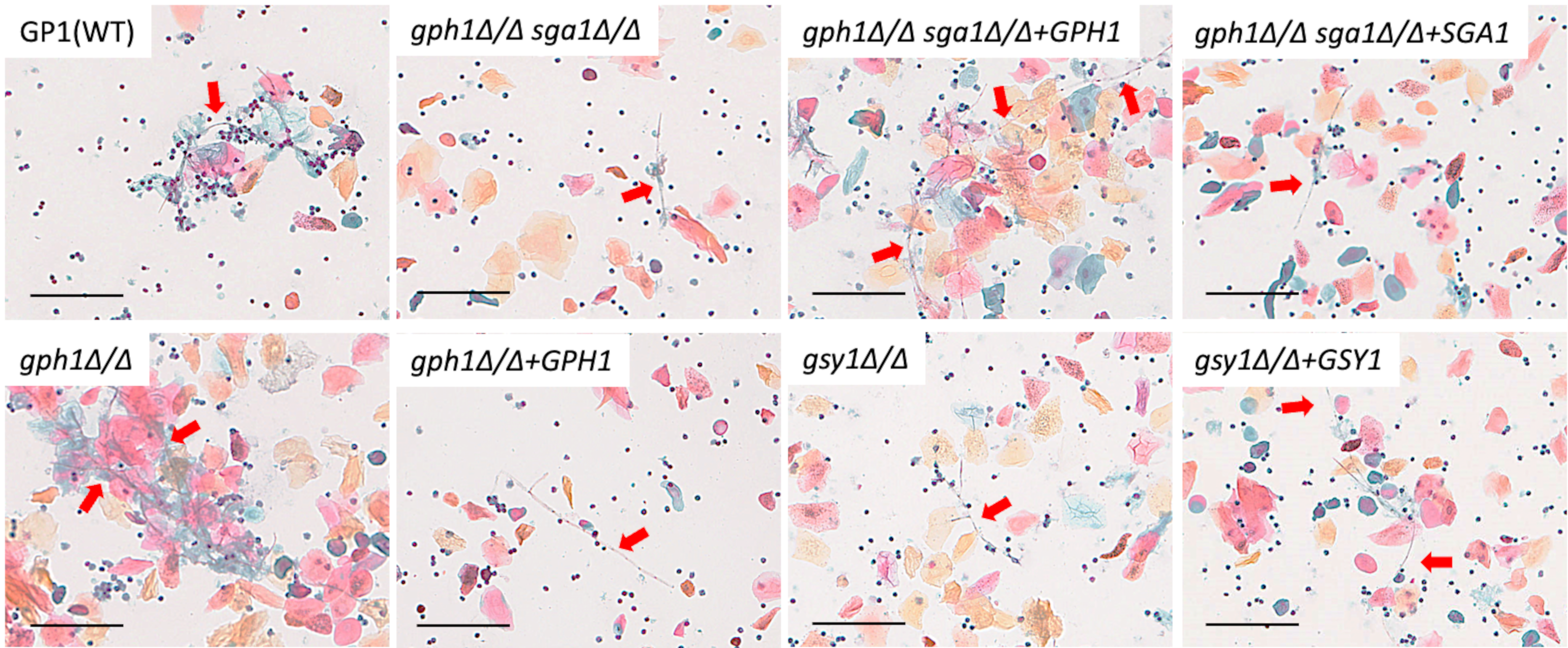

Supplement: FIG S4 [file mbio.00046-23-s0004.tif]
