# Supplementary material for: Glycogen Metabolism in Candida albicans Impacts Fitness and Virulence during Vulvovaginal and Invasive Candidiasis
Source: mBio. 2023 Feb 22;14(2):e00046-23. doi: 10.1128/mbio.00046-23 (PMC10127583; doi:10.1128/mbio.00046-23)
Supplement: TABLE S1 [file mbio.00046-23-s0005.pdf]

| Species | Strain names used | Parent | Genotype | Reference |
|---|---|---|---|---|
| *C. parapsilosis* | CDC317 | N/A | Wild type, reference isolate | (1) |
| *C. glabrata* | CBS138 | N/A | Wild type, reference isolate | (1) |
| *C. krusei* | 4172 | N/A | Wild type, reference isolate | (1) |
| *C. tropicalis* | MYA3404 | N/A | Wild type, reference isolate | (1) |
| *C. dubliniensis* | CD36 | N/A | Wild type, reference isolate | (1) |
| *C. auris* | [429]0382 | N/A | Wild type, reference isolate | (1) |
| *C. albicans* | SC5314 | N/A | Wild type, reference isolate | (2) |
| *C. albicans* | BWP17 | SC5314 | *arg4Δ/Δ his1Δ/Δ ura3Δ/Δ* | (3) |
| *C. albicans* | GP-1 | BWP17 | *arg4Δ/Δ::ARG4 his1Δ/Δ::HIS1 ura3Δ/Δ::URA3* | (4) |
| *C. albicans* | *gph1Δ/Δ* | BWP17 | *gph1Δ::ARG4/gph1Δ::HIS1 ura3Δ/Δ::URA3* | this study |
| *C. albicans* | *gph1Δ/Δ+GPH1* | *gph1Δ/Δ* | *gph1Δ::ARG4/gph1Δ::HIS1 ura3Δ/Δ::URA3-GPH1* | this study |
| *C. albicans* | *gdb1Δ/Δ* | GP-1 | *Δgdb1::FRT+/Δgdb1::FRT+* | this study |
| *C. albicans* | *gdb1Δ/Δ+GDB1* | *gdb1Δ/Δ* | *Δgdb1::FRT+/Δgdb1::FRT+ NEUT5Δ::NAT1-PrGDB1-GDB1-tADH1/NEUT5* | this study |
| *C. albicans* | *sga1Δ/Δ* | BWP17 | *sga1Δ::ARG4/sga1Δ::HIS1 ura3Δ/Δ::URA3* | this study |
| *C. albicans* | *sga1Δ/Δ+SGA1* | *sga1Δ/Δ* | *sga1Δ::ARG4/sga1Δ::HIS1 ura3Δ/Δ::URA3-SGA1* | this study |
| *C. albicans* | *glg1Δ/Δ* | BWP17 | *glg1Δ::ARG4/glg1Δ::HIS1 ura3Δ/Δ::URA3* | this study |
| *C. albicans* | *glg1Δ/Δ+GLG1* | *glg1Δ/Δ* | *glg1Δ::ARG4/glg1Δ::HIS1 ura3Δ/Δ::URA3-GLG1* | this study |
| *C. albicans* | *glg2Δ/Δ* | BWP17 | *glg2Δ::ARG4/glg2Δ::HIS1 ura3Δ/Δ::URA3* | this study |
| *C. albicans* | *glg2Δ/Δ+GLG2* | *glg2Δ/Δ* | *glg2Δ::ARG4/glg2Δ::HIS1 ura3Δ/Δ::URA3-GLG2* | this study |
| *C. albicans* | *gsy1Δ/Δ* | BWP17 | *gsy1Δ::ARG4/gsy1Δ::HIS1 ura3Δ/Δ::URA3* | this study |
| *C. albicans* | *gsy1Δ/Δ+GSY1* | *gsy1Δ/Δ* | *gsy1Δ::ARG4/gsy1Δ::HIS1 ura3Δ/Δ::URA3-GSY1* | this study |
| *C. albicans* | *glc3Δ/Δ* | BWP17 | *glc3Δ::ARG4/glc3Δ::HIS1 ura3Δ/Δ::URA3* | this study |
| *C. albicans* | *glc3Δ/Δ+GLC3* | *glc3Δ/Δ* | *glc3Δ::ARG4/glc3Δ::HIS1 ura3Δ/Δ::URA3-GLC3* | this study |
| *C. albicans* | *gph1Δ/Δ sga1Δ/Δ* | *gph1Δ/Δ* | *gph1Δ::ARG4/gph1Δ::HIS1 ura3Δ/Δ::URA3 sga1Δ::FRT+/sga1Δ::FRT+* | this study |
| *C. albicans* | *gph1Δ/Δ sga1Δ/Δ+GPH1* | *gph1Δ/Δ+GPH1* | *gph1Δ::ARG4/gph1Δ::HIS1 ura3Δ/Δ::URA3-GPH1 sga1Δ::FRT+/sga1Δ::FRT+* | this study |
| *C. albicans* | *gph1Δ/Δ sga1Δ/Δ+SGA1* | *sga1Δ/Δ+SGA1* | *sga1Δ::ARG4/sga1Δ::HIS1 ura3Δ/Δ::URA3-SGA1 gph1Δ::FRT+/gph1Δ::FRT+* | this study |
| *C. albicans* | GP1-GFPy | GP-1 | *NEUT5Δ::*Pr*TEF1-GFPy-tADH1/NEUT5* | this study |
| *C. albicans* | *gph1Δ/Δ*-dTomato | *gph1Δ/Δ* | *gph1Δ::ARG4/gph1Δ::HIS1 ura3Δ/Δ::URA3 NEUT5Δ::NAT1-*Pr*TEF1-dTomato-tADH1/NEUT5* | this study |
| *C. albicans* | *gph1Δ/Δ+GPH1*-dTomato | *gph1Δ/Δ+GPH1* | *gph1Δ::ARG4/gph1Δ::HIS1 ura3Δ/Δ::URA3-GPH1 NEUT5Δ::NAT1-*Pr*TEF1-dTomato-tADH1/NEUT5* | this study |
| *C. albicans* | *gdb1Δ/Δ*-dTomato | *gdb1Δ/Δ* | *Δgdb1::FRT+/Δgdb1::FRT+ NEUT5Δ::NAT1-*Pr*TEF1-dTomato-tADH1/NEUT5* | this study |
| *C. albicans* | *sga1Δ/Δ*-dTomato | *sga1Δ/Δ* | *sga11Δ::ARG4/sga11Δ::HIS1 ura3Δ/Δ::URA3 NEUT5Δ::NAT1-*Pr*TEF1-dTomato-tADH1/NEUT5* | this study |
| *C. albicans* | *sga1Δ/Δ+SGA1*-dTomato | *sga1Δ/Δ+SGA1* | *sga1Δ::ARG4/sga1Δ::HIS1 ura3Δ/Δ::URA3-SGA1 NEUT5Δ::NAT1-*Pr*TEF1-dTomato-tADH1/NEUT5* | this study |
| *C. albicans* | *gsy1Δ/Δ*-dTomato | *gsy1Δ/Δ* | *gsy1Δ::ARG4/gsy1Δ::HIS1 ura3Δ/Δ::URA3 NEUT5Δ::NAT1-*Pr*TEF1-dTomato-tADH1/NEUT5* | this study |

| | | | | |
|---|---|---|---|---|
| *C. albicans* | *gsy1Δ/Δ+GSY1*-dTomato | *gsy1Δ/Δ+GSY1* | *gsy1Δ::ARG4/gsy1Δ::HIS1 ura3Δ/Δ::URA3-GSY1 NEUT5Δ::NAT1*-Pr*TEF1-dTomato-tADH1/NEUT5* | this study |
| *C. albicans* | *glc3Δ/Δ*-dTomato | *glc3Δ/Δ* | *glc3Δ::ARG4/glc3Δ::HIS1 ura3Δ/Δ::URA3 NEUT5Δ::NAT1*-Pr*TEF1-dTomato-tADH1/NEUT5* | this study |
| *C. albicans* | *glc3Δ/Δ+GLC3*-dTomato | *glc3Δ/Δ+GLC3* | *glc3Δ::ARG4/glc3Δ::HIS1 ura3Δ/Δ::URA3-GLC3 NEUT5Δ::NAT1*-Pr*TEF1-dTomato-tADH1/NEUT5* | this study |
| *C. albicans* | *gph1Δ/Δ sga1Δ/Δ*-dTomato | *gph1Δ/Δ sga1Δ/Δ* | *gph1Δ::ARG4/gph1Δ::HIS1 ura3Δ/Δ::URA3 sga1Δ::FRT+/sga1Δ::FRT NEUT5Δ::NAT1*-Pr*TEF1-dTomato-tADH1/NEUT5* | this study |
| *C. albicans* | *gph1Δ/Δ sga1Δ/Δ+GPH1*-dTomato | *gph1Δ/Δ sga1Δ/Δ+GPH1* | *gph1Δ::ARG4/gph1Δ::HIS1 ura3Δ/Δ::URA3-GPH1 sga1Δ::FRT+/sga1Δ::FRT+ NEUT5Δ::NAT1*-Pr*TEF1-dTomato-tADH1/NEUT5* | this study |
| *C. albicans* | *gph1Δ/Δ sga1Δ/Δ+SGA1*-dTomato | *gph1Δ/Δ sga1Δ/Δ+SGA1* | *sga1Δ::ARG4/sga1Δ::HIS1 ura3Δ/Δ::URA3-SGA1 gph1Δ::FRT+/gph1Δ::FRT+ NEUT5Δ::NAT1*-Pr*TEF1-dTomato-tADH1/NEUT5* | this study |

## References


1. Willems HME, Lowes DJ, Barker KS, Palmer GE, Peters BM. 2018. Infect Immun 86:e00527-18.
2. Gillum AM, Tsay EY, Kirsch DR. 1984. Mol Gen Genet 198:179-82.
3. Wilson RB, Davis D, Mitchell AP. 1999. J Bacteriol 181:1868-74.
4. Willems HME, Bruner WS, Barker KS, Liu J, Palmer GE, Peters BM. 2017. Infect Immun 85:e00248-17.
