# Supplementary material for: Glycogen Metabolism in Candida albicans Impacts Fitness and Virulence during Vulvovaginal and Invasive Candidiasis
Source: mBio. 2023 Feb 22;14(2):e00046-23. doi: 10.1128/mbio.00046-23 (PMC10127583; doi:10.1128/mbio.00046-23)
Supplement: TABLE S2 [file mbio.00046-23-s0006.pdf]

| Vector | Reference |
|---|---|
| pLUX | (1) |
| pLUX-GPH1 | this study |
| pLUX-SGA1 | this study |
| pLUX-GLG1 | this study |
| pLUX-GLG2 | this study |
| pLUX-GSY1 | this study |
| pLUX-GLC3 | this study |
| pDUP3 | (2) |
| pDUP3-GFPy | this study |
| pDUP3-dTomato | this study |
| pDUP3-tADH1 | (3) |
| pKE4-GFPy | (4) |
| pKE4-dTomato | (4) |

**References**


1. Goshorn AK, Grindle SM, Scherer S. 1992. Infect Immun 60:876-84.
2. Gerami-Nejad M, Zacchi LF, McClellan M, Matter K, Berman J. 2013. Microbiology (Reading) 159:565-579.
3. Liu J, Vogel AK, Miao J, Carnahan JA, Lowes DJ, Rybak JM, Peters BM. 2022. Microbiol Spectr 10:e0263021.
4. Butts A, DeJarnette C, Peters TL, Parker JE, Kerns ME, Eberle KE, Kelly SL, Palmer GE. 2017. mSphere 2:e00379-17.
