# Supplementary material for: Glycogen Metabolism in Candida albicans Impacts Fitness and Virulence during Vulvovaginal and Invasive Candidiasis
Source: mBio. 2023 Feb 22;14(2):e00046-23. doi: 10.1128/mbio.00046-23 (PMC10127583; doi:10.1128/mbio.00046-23)
Supplement: TABLE S3 [file mbio.00046-23-s0007.pdf]

Primer or RNA name	Sequence (5' to 3')[a]
GPH1_AmpF-KpnI	TCA**GGTACC**GGAAGGTTAGTGGGGAGAGAGAAAT
GPH1_AmpR-SacI	TCA**GAGCTC**CTTTCTCTCCCTTCATTGCCGAG
GPH1_DISF	ATTATTTTCAGGTAACGTTTTTATACAGTTATAGATCTAATTAATTAATTGATAGTGTTGTGATCGTTTCCCAGTCACGACGTT
GPH1_DISR	TAGCTAAGAATTAAAAGTGGTAAAACACAACAACAAAAGATCTTTCAAAAAAATTTCAAGGACCATGTGGAATTGTGAGCGGATA
GPH1_DETF	GGGTTGCTGCTTCCTTGCATGATA
GPH1_DETR	GCTTGTCTCAACCAACGTCTTGG
GPH1_seq1	GACCAACCGACAACAACAACAACTTTTATT
GPH1_seq2	CAGAACCTGGACAAATGATTTCTGCTTTAG
GPH1_seq3	GACCGATTTTGGACTCTCTTCAATGATAGA
GPH1_seq4	AACCGTAAAAGTCAACTGGGATTTGAATTT
GPH1_CC9KO-F	AGATATGCAGACGTACATTGCCCCCCTAATTTCTTCTATAGGTTTTCCCAGTCACGACGT
GPH1_CC9KO-R	CTTTTTTTTTTTCTATTTTTCCCGCGAGTTTTGTTTATCTGTGTGGAATTGTGAGCGGAT
GDB1_CC9KO-F	TCCGCTTAAATTGGAACGGGGGACAAAAAGACAATGACGAGGTTTTCCCAGTCACGACGT
GDB1_CC9KO-R	TATTTTTAATTGTTAGGAATTCCTGTTTGGACTATTGGCAGTGTGGAATTGTGAGCGGAT
crGPH1_up	CCGGAAATGTTGAACTTGCT
crGPH1_down	ATATTCGAATCAAGTTCGGA
crGDB1_up	ACACGACGACGTTTACCGAG
crGDB1_down	TATACGACGTTGACGTAAAG
GDB1_AmpF-KpnI	TCA**GGTACC**GTGGAGGGCTGTTGTAAAGTCTT
GDB1_AmpR-SacI	TCA**GAGCTC**GCTTCCCAATCCACAATCTACACAC
GDB1_DETF	TGAAATGCCAGCACAAAAGCGAACT
GDB1_DETR	GATCCAGGAATGAACGAGGCTTCAT
PrGDB1_INTR	ACTCATGGATCAGCCATCATCAAGC
PrGDB1_OL-F	CCTCGAGGTCGACGGTATCGGTGGAGGGCTGTTGTAAAGTCTTG
GDB1_OL-R	ATTTGCTTAGCATGCACGCGCTAAATTCTGTCTGAACAATCTAAATATCTATATAG
GDB1_seq1	GGATCTACTGTATACAGTACGAAGCACTGG
GDB1_seq2	GGGACTTTATACACTAATGTGCCCTCAAAG
GDB1_seq3	GACGGTATTCAAACTCACGTGTTGGATAA
GDB1_seq4	AATTGTCTGCCAAGACTTTCAATGGGTTTA
GDB1_seq5	AGATATAATGCAAGAACAGGTAACGGTTGG
GDB1_seq6	CTTATTATGGGTATTGCTTATGAAGCAGCC
GDB1_seq7	GGTGGTAATCAATTCAATTGTGGTACTTGG
GDB1_seq8	GACCAGAATGGGTTTGGAATACTGGATATT
SGA1_AmpF-KpnI	TCA**GGTACC**GCTGTTGCATGTTGTCAACTTGAAT

SGA1_AmpR-SacI	TCA**GAGCTC**TATGGGATCCCTTATCACGTGCCAC
SGA1_DISF	ATCGTTATACCTTCCTATAGATTAGACTTTTTATTACATTTTATTTTAGAACTTCACACTAAGTCGTTTTCCCAGTCACGACGTT
SGA1_DISR	CAGTATTCAATTGTTTTAAAAAACACTATACAAAATTAATCAATAAAAAACAACTAAAGCTAATTTGTGGAATTGTGAGCGGATA
SGA1_DETF	AGCATCACCGTCAACTTCAAATCCT
SGA1_DETR	CAGGGTATCGACCTAATGCTACTCC
SGA1_seq1	ATCACGTGCCACTTATTCTTTCATTTAACG
SGA1_seq2	TATCTTCAGGGTATCGACCTAATGCTACTC
SGA1_seq3	GGTTCACCTAAATTTTCCAAGTTGTCAACA
crSGA1-up	GTTGCTGTGGATAGTAGAGA
crSGA1-down	TGAGTTATTAGATCGCTCAG
SGA1_CC9KO-F	TATACATTGTCATTAATGGAGTAACTAGTTGATTGGTTACGGTTTTCCCAGTCACGACGT
SGA1_CC9KO-R	AAGAAGACGAGAAAATATTGTTATCTGATCAAGAAATTGAGTGTGGAATTGTGAGCGGAT
SGA1_AmpR-v2	ATGGACAAACCAGTCAAGATTCAGC
GLG1_AmpF-StuI	TCA**AGGCCT**AATACGCTCCCACTGCAGCTTATAT
GLG1_AmpR-SacI	TCA**GAGCTC**AGTTCGTAGAGTGAAAGTGCACAGA
GLG1_DISF	ATCCATTTATCCCACAGTTTTCCAGAAAAAAATACACACACATATCTATACAATAAGTATCTCCGGTTTTCCCAGTCACGACGTT
GLG1_DISR	TAAGGTAATTACTACGGAAATCTTTTCTGATATCTTCTTTCATATACTATTGATTCTTATGAATTTGTGGAATTGTGAGCGGATA
GLG1_DETF	GGATCAGAGGTATCAAGTGGTGGTT
GLG1_DETR	ACTTATGATTTGAAGAGACCGGAATTGGAC
GLG1_seq1	TGTTTCATTTCTCCCCTGTCGTTGT
GLG1_seq2	ACCCTAATGTATCTTTTGATGGTGCTGATC
GLG1_seq3	AATTCAACCTAGCATCGATGCCATG
GLG2_AmpF-KpnI	TCA**GGTACC**CACAAGCTCGCAAAATAGTTTTGTCC
GLG2_AmpR-SacI	TCA**GAGCTC**GAACAAACCAAATCAATGCCAGGTC
GLG2_DISF	ACAAATCTATACAACAACTACATCTCCACTACTTCTTTTTCTACTTATATATTCATTACAATCAGGTTTCCCAGTCACGACGTT
GLG2_DISR	TCTTTCAAATCATTCTTAAGCCCATAATTTTATTCCAAAGAAAAACTAGTGGCATTCTTGTCAACTGTGGAATTGTGAGCGGATA
GLG2_DETF	TTGGTGCTGCTTCTGATTCTGGATG
GLG2_DETR	TTGGAAAACACGAGTAGGCTGTCTT
GLG2_seq1	TAATCAAATCAAGTTCACCTTCAACCTCGC
GLG2_seq2	TGGAGGCGATCAAGGATTGTTTAATGAATA
GSY1_AmpF-KpnI	TCA***GGTACC***CATGACCCAATGTTGGCTTTATTAGT
GSY1_AmpR-SacI	TCA***GAGCTC***AGAAAGGCGTTAAGAACCATTCAGTC
GSY1_DISF	TCGTTTTGTTTCATTTTCCCACCGTCCCCCCTTTAGTTTTTGTCCACATTTAATTTAGAAGAACAGTTTCCCAGTCACGACGTT
GSY1_DISR	GTAAGAAACCTATACATATGTATATAATTTATAAACAAGCATATAATATTGGGCAAAAATTAAGTTGTGGAATTGTGAGCGGATA
GSY1_DETF	TACCCCTGATCATGACCTGGAAACC
GSY1_DETR	AGGTTCGTAGTAGGATGGGAACACA

GSY1_seq1	CTCTGACTTGAGATTGCCTAATTAGTCACC
GSY1_seq2	TTCAAGACTGGATCAGTACTGTCATCAACC
GSY1_seq3	CCAGCACACAAGTATCTTCCCAATAATGTT
GLC3_AmpF-AvrII	TCA**<u>CCTAG</u>**GGGCTGGTGATGTTGATGTTGTTGTT
GLC3_AmpR-SacI	TCA**<u>GAGCTC</u>**GTTAATCAAATCTTGATTGCGCCGC
GLC3_DISF	ATCGTTATACCTTCCTATAGATTAGACTTTTTATTACATTTTATTTTAGAACTTCACACTAAGTCGTTTTCCCAGTCACGACGTT
GLC3_DISR	CAGTATTCAATTGTTTTAAAAAACACTATACAAAATTAATCAATAAAAAACAACTAAAGCTAATTTGTGGAATTGTGAGCGGATA
GLC3_DETF	CTACTCCGGAACCAACTATTGGTAGT
GLC3_DETR	CGAGTCTTCCATGACCACCTAAATCT
GLC3_seq1	GGTACCAGAGCTCGTTAATCAAATCTTGAT
GLC3_seq2	ACGGATTAATTTATGAAGGGCAATACCTCT
GLC3_seq3	TTAATTCATCAGGGGTACCATATCGAGATG
HIS1_INTF2	ACTGTATCCTCTTCTGTCCCC
HIS1_INTR2	CGACCATATGGGAGAGCTCCC
ARG4_INTF2	AAGCTAGTGTGGAAAGAAGAG
ARG4_INTR2	AATGACTGAATTATGTCGGTC
LUXINT_DETF	CTGACCTTTAGTCTTTCCTGC
LUXINT_DETR	CAGTAGTACTTGTTGTTGTATCG
N5ADH1_up-UNIVOL-F	CCTCGAGGTCGACGGTATCG
N5ADH1_up-UNIVOL-R	CGATACCGTCGACCTCGAGG
ADH1t-UNIV-OL-F	CGCGTGCATGCTAAGCAAAT
NAT1_INTF	CCCAGATGCGAAGTTAAGTGCG
NEUT5L_homology-F	GCAGATATGAGATAAAAGTTTTAAAGGACAAGAAAAGG
NEUT5L_homology-R	ATCTCTAATAATTGCAATTGCAATTGCTTCACATA
crNEUT5pDUPup	GTAGTAAGACAATATGACTT
PrTEF1-F-ClaI	TCA**<u>ATCGAT</u>**GACGGCCAGTCCGTAATACGA
tADH1-R-SpeI	TCA**<u>ACTAGT</u>**GAAAACTTGAAACTTGAAAACACCG

[a]Engineered restriction enzyme sites are highlighted in bold text and underlined.
